# Prediction of 8-state protein secondary structures by a novel deep learning architecture

**DOI:** 10.1186/s12859-018-2280-5

**Published:** 2018-08-03

**Authors:** Buzhong Zhang, Jinyan Li, Qiang Lü

**Affiliations:** 1 School of Computer Science and Technology, Soochow University, Suzhou, China; 2 School of Computer and Information, and the University Key Laboratory of Intelligent Perception and Computing of Anhui Province, Anqing Normal University, Anqing, 246011 China; 3 Advanced Analytics Institute, Faculty of Engineering and IT, University of Technology Sydney, Broadway, NSW 2007, Sydney, PO Box 123 Australia

**Keywords:** Protein secondary structures, Q8 prediction, Local block, Deep learning

## Abstract

**Background:**

Protein secondary structure can be regarded as an information bridge that links the primary sequence and tertiary structure. Accurate 8-state secondary structure prediction can significantly give more precise and high resolution on structure-based properties analysis.

**Results:**

We present a novel deep learning architecture which exploits an integrative synergy of prediction by a convolutional neural network, residual network, and bidirectional recurrent neural network to improve the performance of protein secondary structure prediction. A local block comprised of convolutional filters and original input is designed for capturing local sequence features. The subsequent bidirectional recurrent neural network consisting of gated recurrent units can capture global context features. Furthermore, the residual network can improve the information flow between the hidden layers and the cascaded recurrent neural network. Our proposed deep network achieved 71.4% accuracy on the benchmark CB513 dataset for the 8-state prediction; and the ensemble learning by our model achieved 74% accuracy. Our model generalization capability is also evaluated on other three independent datasets CASP10, CASP11 and CASP12 for both 8- and 3-state prediction. These prediction performances are superior to the state-of-the-art methods.

**Conclusion:**

Our experiment demonstrates that it is a valuable method for predicting protein secondary structure, and capturing local and global features concurrently is very useful in deep learning.

**Electronic supplementary material:**

The online version of this article (10.1186/s12859-018-2280-5) contains supplementary material, which is available to authorized users.

## Background

A protein is a linear chain of amino acids connected by peptide bonds. The primary structure of a protein is just the amino acid sequence ordered in the polypeptide chain. Repeated regular conformations on the polypeptide chain are called the secondary structures of proteins. From the secondary structures, a protein can be folded into a stable three-dimensional structure, which is called the tertiary structure of a protein. Although a protein’s structure is largely determined by its amino acid sequence [1], advanced studies show that accurate prediction of tertiary structures from sequences is a challenging problem currently with poor performance. The prediction of protein secondary structures from sequences is then considered as an intermediate problem bridging the gap between the primary sequences and tertiary structure prediction.

Protein secondary structures are traditionally characterized as 3 general states: helix (H), strand (E), and coil (C). From these general three states, the DSSP program [2] proposed a finer characterization of the secondary structures by extending the three states into eight states: 3_10_ helix (G), *α*-helix (H), *π*-helix (I), *β*-stand (E), bridge (B), turn (T), bend (S), and others (C). Prediction of the three states from protein sequences (i.e., the Q3 prediction problem) has been intensively investigated for decades using many machine learning methods, including the probability graph models [3, 4], support vector machines [5, 6], hidden Markov models [7, 8], artificial neural network [9–12], and bidirectional recurrent neural network(BRNN) [13–16].

Recently, the focus of secondary structure prediction has been shifted from Q3 prediction to the prediction of 8-state secondary structures, due to the fact that a chain of 8-state secondary structures contains more precise structural information for a variety of applications. The prediction of the 8 states of secondary structures from protein sequences is called a Q8 prediction problem. The Q8 problem is much more complicated than the Q3 problem. Because it is considerably more complicated than Q3 prediction, deep learning methods have been applied. For example, SC-GSN network [17], the bidirectional long short-term memory (BLSTM) method [18, 19], the deep conditional neural field [20], DCRNN [21], the next-step conditioned deep convolutional neural network(CNN) [22] and Deep inception-inside-inception (Deep3I) network [23] have been widely explored.

Protein secondary structures are not confined to only adjacent residues, but also involved with long-range residue contacts. Many literature computational methods have considered these biological facts to combine both local and long-range contact information. DeepCNF [20] is a Deep Learning extension of Conditional Neural Fields, which combines the advantages of both conditional neural fields and deep convolutional neural networks. DCRNN [21], comprised of a multi-scale convolutional layer linked by three stacked bidirectional recurrent network layers, uses CNN to obtain the local information and BRNN to obtain long-range contact information. An ensemble of ten independently trained DCRNN has achieved a 69.7% accuracy on the CB513 benchmark data set. Next-Step Conditioned CNN [22] combines the previous labels to the current input to remember the former information like RNN. It further improves the prediction performance to a 70.3% accuracy. When trained under an ensemble learning framework, it has achieved a 71.4% accuracy, representing the newest state-of-the-art performance of the Q8 prediction problem. Based on the Google Inception network [24], a Deep inception-inside-inception (Deep3I) network [23], named MUFOLD-SS which are mainly constructed by CNNs and residual networks(Resnet) [25], is proposed. MUFOLD-SS uses inception-inside-inception and Resnet to enhance the performance of capturing long-range contact information in sequences. MUFOLD-SS has been evaluated for the Q8 and Q3 prediction performance on the CB513, CASP10, CASP11 and CASP12 datasets. Very recently, Port 5 [16] assembling seven BRNNs have achieved 73% and 84.2% of Q8 and Q3 prediction on 3315 protein sequences respectively.

In this study, we propose to use a convolutional, residual, and recurrent neural network (CRRNN) for both Q8 and Q3 secondary structure prediction. Firstly a local block comprising of one-dimensional CNNs and the original input combines local features and original sequence information. After local block filtering, the sequences are fed to a bidirectional recurrent neural network (BRNN) containing gated recurrent units (GRU) [26]. This architecture of BRNN can model the sequence structure and can capture long-range dependencies of the residues. The BRNN is a three-layer stacked structure with residual connections [25] linked to the interval BRNN layer. To reduce the high-dimensionality of hidden-layer input, a 1D convolutional filter with one kernel [24] is used along with the residual connection. The multi-perception and softmax layer for the final classification are then connected. We used 12,148 sequences to train the model and tested its performance on the benchmark data sets CB513, CASP10, CASP11 and CASP12. We also trained ten individual model and ensemble them as a integrated model named as eCRRNN. The prediction results have demonstrated that the deep network has better generalization performance in comparison with the best existing method. The superior performance is mainly attributed to: (i) The local block can integrate both local features and the original sequence information; the 1D CNN rather than 2D CNN is used for processing sequence data in local block. (ii) A novel deep learning model, CRRNN for sequence to sequence learning is proposed; The model parameters are evaluated and 1D convolutional filter with one kernel is used for dimensionality reduction.

## Materials

### Datasets

A hybrid training set and five independent test datasets were used in this study. The training data is named TR12148 which consists of 12,148 polypeptide chains from the integration of the existing benchmark datasets TR5534 and TR6614. TR5534 was prepared by [17] that contains 5534 proteins. This benchmark dataset has been used to train the deep learning models including SC-GSN [17], DCRNN [21], and conditioned CNN [22]. In fact, TR5534 was derived from the 6128 proteins of the CB513 dataset after sequence identity reduction. Dataset TR6614 contains 6614 non-homologous sequences produced using the PISCES Cull PDB server [27]. Protein sequences in TR6614 have a similarity less than 25%, a resolution better than 3.0Å and an R factor of 1.0. The redundancy with test datasets was removed using cd-hit [28]. A detailed sequences list of TR6614 is given in Additional file 1 in supplemental information. We randomly selected 248 proteins as a validation dataset (VR248) and 240 proteins as test dataset (TS240) from TR12148, respectively, and used the remaining 11,700 proteins for training. The 3D structure files were downloaded from the RCSB Protein Data Bank (PDB).

Four public test datasets (named CB513, CASP10, CASP11, and CASP12) were used to evaluate the Q8 and Q3 performance of our proposed model. CB513 is from [17]. CASP10, CASP11, and CASP12 are from the “Protein Structure Prediction Center”. CASP10 contains 123 domain sequences extracted from 103 chains; CASP11 contains 105 domain sequences extracted from 85 chains; and CASP12 contains 40 chains. The total residues of the sequences from CASP10, CASP11, CASP12 and CB513 are 22041, 20498, 10526 and 87041 respectively. More details of the Q8 secondary structures in these datasets are listed in Table 1.

**Table 1:** Training and test data used in our work

Label	Types	TR6614		TR5534		CB513		CASP10		CASP11		CASP12	
		Count	%	Count	%	Count	%	Count	%	Count	%	Count	%
H	*α*-helix	517653	0.352	405560	0.345	26143	0.309	6544	0.297	6330	0.309	3550	0.337
B	*β*-bridge	15321	0.010	12096	0.010	1180	0.014	227	0.010	221	0.011	113	0.011
E	*β*-strand	321156	0.218	255887	0.218	17994	0.212	5225	0.237	5089	0.248	2223	0.211
G	3_10_helix	55994	0.038	46019	0.039	3132	0.037	797	0.036	716	0.035	320	0.030
I	*π*-helix	281	0	209	0	30	0	5	0	0	0	0	0
T	Turn	160753	0.109	132980	0.113	10008	0.118	2811	0.128	2299	0.112	1164	0.111
S	Bend	118800	0.081	97298	0.083	8310	0.098	1780	0.081	1751	0.085	955	0.091
L	Coil	282584	0.192	225493	0.192	17904	0.211	4652	0.211	4092	0.200	2201	0.209
All		1472542		1175542		84701		22041		20498		10526	

TR12148 is a dataset merging TR5534 and TR6614, and it contains 2,976,315 residues. The sequence lengths of the proteins in TR6614 range from 60 to 700 and the length range of the proteins in TR5534 is from 50 to 700. Sequence lengths of the proteins in the test datasets are capped at 700 as well. If the length of a sequence from the test datasets is longer than 700, the sequence is splitted into two sequences. The 700-residue length cut-off was chosen to provide a good balance between efficiency and coverage, given that the majority of the protein chains are shorter than 700 residues.

### Input features

Four types of features, including a position-specific scoring matrix (PSSM), protein coding features, conservation scores, and physical properties, are used to characterize each residue in a protein sequence. To generate a PSSM, we ran PSI-Blast [29] to search the NCBI non-redundant database through three iterations with E-value=0.001. The physical property features [30] have been previously used for protein structure and property prediction [19, 31]. These physical properties are: steric parameters (graph-shape index), polarizability, normalized van der Waals volume, hydrophobicity, isoelectric point, helix probability, and sheet probability. These specific values were downloaded from Meiler’s study [30]. To ensure the network gradients decrease smoothly, these above 27 features were normalized by logistic function.

The 1-dimensional conservation score was computed by the method [32](1),

1 $$ R=\log20+ \sum\limits_{i=1}^{20}Q_{i}\log Q_{i}  $$

Residue conversion was conducted according to amino acid frequency distribution in the corresponding column of a multiple-sequence alignment of homologous proteins. The score information in the PSSM was calculated from this probability. Residue score in the *i*-th column was calculated as follows [33]:

2 $$ S_{i}=\left[\ln(Q_{i}/P_{i})\right]/\lambda_{u}.  $$

where *Q*_*i*_ is a predicted probability that a properly aligned homologous protein has amino acid *i* in that column, *P*_*i*_ is the background probability [29], and *λ*_*u*_=0.3176. *Q*_*i*_ is defined as *Q*_*i*_= exp(*S*_*i*_∗*λ*_*u*_)∗*P*_*i*_.

The commonly used protein coding is an orthogonal coding. As Zhou’s [17] scheme, the 22-dimensional coding vector is a sparse one-hot vector, only one of 22 elements is none-zero and a zero vector is no use for gradient optimization. Like description by [21], we adopted an embedding operation from natural-language processing to transform sparse sequence features into a denser representation. This embedding operation was implemented as a feed-forward neural network layer with an embedding matrix mapping a sparse vector into a denser 22-dimensional vector.

In our scheme, one residue is represented by 50-dimensional features (20-dimensional PSSM, 7-dimensional physical properties, 1-dimensional conservation score and 22-dimensional protein coding information). The secondary structure labels are generated by DSSP [2]. Similar to Zhou’s method [17], proteins shorter than 700 AA were padded with all-zero features and the corresponding outputs are labeled with “NoSeq”. The advantage of padding these proteins is to enable the training of the model on GPU in batches.

## Methods

**Fig. 1:**
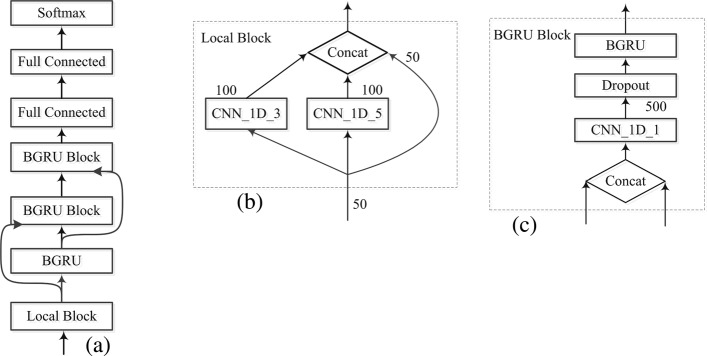
**a** CRRNN overall architecture. **b** A local block comprising of two 1D convolutional networks with 100 kernels, and the concatenation (Concat) of their outputs with the original input data. **c** the BGRU block. The concatenation of input from the previous layer and before the previous layer is fed to the 1D convolutional filter. After reducing the dimensionality, the 500-dimensional data is transferred to the next BGRU layer

As illustrated in Fig. 1, our CRRNN model consists of four parts: a local block, three stacked bidirectional gated recurrent unit (BGRU, or BGRU block) layers, two residual connections, and two fully-connected layers. The local block capture local sequence features and feeds them to the first BGRU layer, and the residual network transfers data to the subsequent BGRU layers. In the BGRU block, two types of input data are concatenated and fed to the next BGRU layer. At the end of the fully connected layer, the softmax activation outputs the predicted results in either the 8- or 3-state category.

### Local block

Extracting information from protein sequences by convolutional neural network has fast progressed [17, 20–22]. The application of the convolution operator is dependent upon input dimensionality [34]. Two-dimensional kernels are often used in a 2D spatial convolutional operator, whereas a 1D convolutional network is usually used for processing sequences. In the 1D domain, a kernel can be viewed as a filter capable of removing outliers to filter data or act as a feature detector. Here, we used a 1D CNN to model the local dependencies of adjacent amino acids. Given the sequence data

3 $$ X=(x_{1},x_{2},x_{3}{\dots}x_{t-1},x_{t},x_{t+1}{\dots}x_{n}),  $$

where *x*_*i*_=(*x*_*i*1_,*x*_*i*2_,…*x*_*ij*_,…*x*_*im*_) is a feature vector of the *i*th residue. Residue *x*_*i*_ is context-dependent and strongly reliant on forward and backward information; however, the value space of feature *x*_*ij*_ might differ from *x*_*ik*_. Overall, residue orientation is convoluted by the 1D CNN:

4 $$ h_{i}=f(W*x_{i:i+k-1}+b) \\  $$

where “*” denotes the convolutional operation, and *k* represents the kernel size. Considering that the minimum length of the second structure, the kernel sizes of CNN in local block are set to three and five. One-hundred filters were used separately, and a rectified linear unit function activates the network output. To capture more structure information, the original input data is concatenated with the convolutional network output. Compared with the kernel size of 7, 11 [21] and 9 ×24 [22], our network parameters were smaller and they could effectively capture the local information.

### BGRU and BGRU block

Protein structures are affected largely by long-range interactions between residues. Recurrent neural network (RNN) can model large-distance dependencies between amino acids. At a given time *T*=*t*, the recurrent neural network can remember information from past input, *x*_1_,*x*_2_,*x*_3_…*x*_*t*−1_, and current input *x*_*t*_. However, the output, *y*_*t*_, might depend upon the contextual protein sequence. The BRNN [35] combines a RNN that moves forward through time beginning from the start of the sequence along with another RNN that moves backward through time beginning from the end of the sequence. In the BRNN, increased input over time is represented by $\overrightarrow {f}(x_{1},x_{2},x_{3},{\dots },x_{t-1})$, and the decreased input over time is represented by $\overleftarrow {f}(x_{t+1},{\dots },x_{n})$. Compared to RNN, the BRNN is more suitable for context-related applications, and its performance is better than unidirectional RNN.

The depth of a RNN makes the network difficult to train because of an exploding or vanishing gradient [36]. Long short-term memory (LSTM) [37], which consists of a variety of gate structures (forgotten gate, input gate, output gate and memory cell) can overcome with the vanishing gradient problem. Compared with a LSTM, gate recurrent units (GRU) achieved comparable performance, and required fewer parameters [36]. The details of GRU is described by the following formula (5):

5 $$ \begin{array}{l} r_{t}=\sigma(W_{xr}x_{t}+W_{hr}h_{t-1}+b_{r}) \\ z_{t}=\sigma(W_{xz}x_{t}+W_{hz}h_{t-1}+b_{z}) \\ \tilde{h_{t}}=tanh\left(W_{xh}x_{t}+W_{hh}\left(r_{t}\bigodot h_{t-1}\right)+b_{h}\right) \\ h_{t}=z_{t}\bigodot h_{t-1}+(1-z_{t})\bigodot\tilde{h_{t}} \\ \end{array}  $$

where *σ* is the sigmoid function, $\bigodot $ represents an element-wise multiplier. $r_{t}, z_{t}, \tilde {h_{t}}$ and *h*_*t*_ are the reset gate, update gate, internal memory cell activation vectors and output, respectively. We construct three BGRU layers in the CRRNN model. When the forward-computed result *F*_*t*_ is merged with the backward result, *B*_*t*_, merging computation in the first GRU layer is concatenated, and the others are summed, as formula (6):

6 $$ \begin{array}{l} O^{1}_{t}=Concat(F_{t},B_{t}) \\ O^{2,3}_{t}=F_{t}+B_{t} \\ s.t. ~ F_{t}=(\overrightarrow{h_{1}},\overrightarrow{h_{2}},{\dots},\overrightarrow{h_{t}}),\\ ~ ~ ~ ~ ~ B_{t}=(\overleftarrow{h_{t}},\overleftarrow{h}_{t+1},{\dots},\overleftarrow{h_{n}})\\ \end{array}  $$

In first BRNN layer, 250 units were used in the unidirectional RNN, and the dimensionality of the output was 500. In the 2nd and 3rd layer, 500 units were used in the unidirectional RNN. Based on the improved performance of the CNN model [25] using additive identity shortcuts between the outputs of the lower layers and the inputs to higher layers, which improved information flow throughout the network, Fig. 1c shows how we introduce this process into recurrent neural network. $h_{t}^{l}$ is the previous layer output and $h_{t}^{l-1}$ is the previous layer input. *I*_*t*_, the concatenation of them will be fed to current hidden layer,

7 $$  \begin{array}{l} I_{t}=Concat\left(h_{t}^{l},h_{t}^{l-1}\right) \\ I_{t}^{\prime}=f\left(W*I_{t}\right)\\ \end{array}  $$

To avoid the explosion caused by feature concatenation of the input from the previous layer, the BGRU block used the 1D CNN with one kernel to control the high dimensionality. Concatenating operation is not as same as the summing operation used in residual network, for it can reserve more information.

### Implementation details

In our experiments, an Adam optimizing function was used for training the entire network of the default setting parameters. The default learning rate was initially set at 0.0004 with a decreasing step 0.0001, whereas the validation accuracy did not increase after more than 10 epochs. The learning-rate threshold was set to 0.0001. A cross-entropy loss function was used to train the model. Weight constraint of dropout (p = 0.5) used to avoid overfitting were applied to the output filters before advancing to the next BGRU layer. The algorithm was enforced to complete when validation accuracy stopped increasing. When the model had iterated about 130 epochs, it converged and predictive performance stabilized. Our model was implemented in Keras, which is a publicly available deep-learning software. Weights in the CRRNN were initialized using default values, and the entire network was trained on a single NVIDIA GeForce GTX 1080 Ti GPU with 12GB memory.

## Results and discussion

### Performance for Q8 and Q3 prediction

Our model, which was trained individually ten times using the TR12148 dataset, achieved a 73.3 ±0.4% accuracy on the TS240 test set. As an individual model, we performed validation on the CB513 benchmark and achieved a 71.4 ±0.2% accuracy, competitively matching that of the state-of-the-art method using the NCCNN ensemble model [22] and 1.1% higher than the NCCNN single model. The single model of NCCNN was iterated at least 1000 epochs while our model converged after only 130 epochs. We also compared our model with other representative methods, such as MUFOLD-SS [23], DCRNN [21], DeepCNF [20], and GSN [17], and BLSTM [18].

Except that MUFOLD-SS are trained using 9000 proteins, most of them are trained on TR5534. We did re-implement Conditioned CNN and DCRNN and used TR12148 as the training data. As some errors were occurred in the re-implemented 2D CNN, we replaced 2D CNN with 1D CNN. The performance by the re-implemented DCRNN exceeded the original results. The performance by the re-implemented NCCNN is weaker than the original results. Details of precision and recall are shown in Tables 2 and 3. The overall performance is shown in Table 4. DCRNN2 was re-implemented by us and trained on TR12148.

**Table 2:** Q8 predictive precision of individual secondary structures from CB513

Q8 Label	CRRNN	NCCNN	MUFOLD-SS	DCRNN2^a^	DCRNN	DeepCNF
H	**0.86**	0.841	0.855	0.863	0.832	0.849
B	0.466	**0.676**	0.571	0.571	0.554	0.433
E	**0.797**	0.767	0.764	0.768	0.753	0.748
G	0.466	**0.487**	0.413	0.419	0.429	0.49
I	0	0	0	0	0	0
T	0.556	**0.577**	0.572	0.562	0.559	0.53
S	0.494	**0.548**	0.522	0.509	0.518	0.487
L	**0.603**	0.565	0.586	0.571	0.573	0.571

^a^Data is generated by our experiment

Boldface numbers indicate best performance

**Table 3:** Recall of individual secondary structures is compared on CB513 for Q8 prediction

Q8 Label	CRRNN	NCCNN	MUFOLD-SS	DCRNN2^a^	DCRNN	DeepCNF
H	0.926	**0.932**	0.920	0.920	0.933	0.904
B	**0.081**	0.041	0.071	0.003	0.026	0.026
E	0.831	0.821	0.815	**0.841**	0.828	0.833
G	**0.371**	0.285	0.364	0.359	0.252	0.26
I	0	0	0	0	0	0
T	**0.555**	0.524	0.549	0.539	0.522	0.528
S	**0.332**	0.24	0.290	0.258	0.249	0.255
L	0.658	0.69	0.662	0.658	0.652	0.657

^a^Data is generated by our experiment

Boldface numbers indicate best performance

**Table 4:** A comparison of the Q8 accuracy(%) on CB513, CASP10, CASP11 and CASP12 between CRRNN and other state-of-the-art methods

method	CB513	CASP10	CASP11	CASP12
GSN	66.4	-	-	-
BLSTM	67.4	-	-	-
DeepCNF	68.3	71.8	71.7^b^	0.694^b^
DCRNN	69.7	-	-	-
DCRNN2^a^	70.4	73.9	71.2	68.8
NCCNN	70.3	-	-	-
NCCNN^a^	71.4	-	-	-
MUFOLD-SS^b^	70.5	74.2	71.6	69.5
CRRNN	71.4 ±0.2	73.8 ±0.5	71.6 ±0.7	68.7 ±0.8
eCRRNN^a^	74	**76.3**	**73.9**	**70.7**

^a^indicates ensemble model

^b^Data is generated by our experiment

Boldface numbers indicate best performance

For all of these methods, their prediction accuracies on the CASP10 dataset are higher than on the other datasets, and the accuracies on the CASP12 dataset are lower. One reason is that the profiles of CASP10 is extracted from the NCBI NR database which represent the sequences more precisely. CASP12 contains more hard cases and the PSSM profiles are not as good as those in CASP10 or CB513.

Tables 2 and 3 show the model performance on individual secondary structures. F1-score, which corresponds to the harmonic means of precision and recall, is also compared in Table 5. Macro _F1 [38] represents the un-weighted mean of all the categories, whereas micro_F1 represents the averages of global total true positives; therefore, this indicator has the same value as the accuracy.

8 $$  \begin{array}{l} F1 = \frac{2 * (precision * recall)}{(precision + recall)} \\ macro\_F_{1}= \frac{1}{n}\sum\limits_{i=1}^{n}{F_{1i}} \end{array}  $$

**Table 5:** F1 score of individual secondary structure labels using CB513

Q8 Label	CRRNN^a^	CRRNN	NCCNN^a^	NCCNN	MUFOLD-SS	DCRNN2	DCRNN^a^	DeepCNF
H	**0.903**	0.892	0.889	0.884	0.886	0.891	0.880	0.876
B	0.138	**0.139**	0.089	0.077	0.000	0.006	0.050	0.049
E	**0.834**	0.814	0.805	0.793	0.789	0.803	0.789	0.788
G	**0.463**	0.413	0.374	0.360	0.387	0.387	0.317	0.340
I	0	0	0	0	0	0	0	0
T	**0.594**	0.555	0.565	0.549	0.561	0.550	0.540	0.529
S	**0.433**	0.397	0.343	0.334	0.373	0.342	0.336	0.335
L	**0.660**	0.629	0.631	0.621	0.622	0.611	0.610	0.611
macro-F1	**0.503**	0.480	0.462	0.452	0.452	0.449	0.440	0.441
micro_F	**0.74**	0.714	0.714	0.704	0.705	0.704	0.697	0.683

^a^indicates ensemble model

Boldface numbers indicate best performance

The F1 score related to individual secondary structure for our model exceeded those by the other methods, indicating that our model exhibited better predictive ability. The macro_F1 score of our model was also better than those by the other methods.

To validate the generalization capability of our model, independent test datasets CASP10, CASP11, and CASP12 were used. The performance results are reported in Table 4. The performance for CASP10, CASP11, and CASP12 by NCCNN were not supplied.

By the same way as [20], we mapped 8-state labels to 3-state labels: H(8-state) was mapped to H(3-state), E(8-state) was mapped to E(3-state) and others (8-state) were mapped to C(3-state). Q3 predictive performance was compared with those by DCRNN and DeepCNF on Table 6. The Q3 accuracy on the CB513 dataset was 85.3 ±0.4%, which was 1.5% higher than the state-of-the-art methods [21]. The predictive accuracy of our model on CASP10, CASP11 and CASP12 were 86.1 ±0.6%, 84.2 ±0.5% and 82.6 ±1.2% respectively, and most of these were higher than the compared methods.

**Table 6:** Q3 accuracy(%) comparison on CB513 and CASP datasets

Method	CASP10	CASP11	CASP12	CB513
PSIPRED	81.2	80.7	80.5^a^	79.2
JPRED	81.6	80.4	78.8^a^	81.7
DeepCNF	84.4	84.7	83.2^a^	82.3
DCRNN	-	-	-	84
NCCNN	-	-	-	-
MUFOLD-SS^a^	84.3	82.3	81.1	82.7
CRRNN	86.1 ±0.6	84.2 ±0.5	82.6 ±1.2	85.3 ±0.4
eCRRNN	**87.8**	**85.9**	**83.7**	**87.3**

^a^Data is generated by our experiment

Boldface numbers indicate best performance

**Fig. 2:**
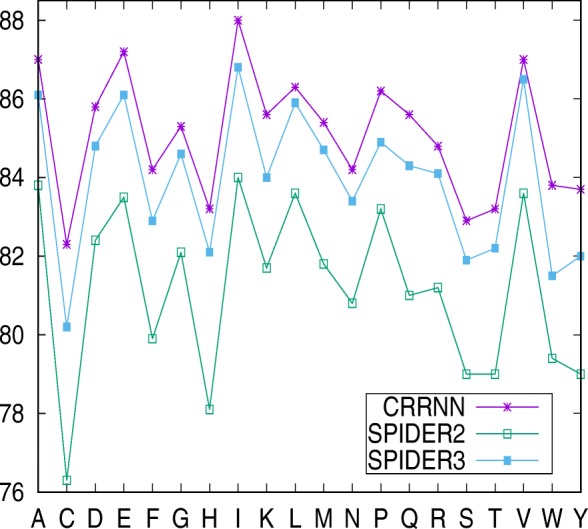
The accuracy of 3-state secondary structure prediction for individual amino acids as compared with CRRNN, SPIDER3 and SPIDER2 on the TS1199 dataset

Another newest Q3 prediction tool SPIDER3 [19] using a two-layered BLSTM was proposed, wherein H, G, and I (8-state) are mapped to H (3-state), E and B (8-state) are mapped to E, and others (8-state) are mapped to C. Similarly, we trained our model and tested it on the TS1199 dataset [19], achieving 85.5% accuracy, which was higher than SPIDER3 (84.5%) and SPIDER2 (81.8%). Figure 2 compares the accuracy of secondary structure prediction at individual amino acid levels with SPIDER3 and SPIDER2, indicating higher accuracies than both at 82%.

### Ensemble learning and case study

In order to further evaluate the model generalization capability, an ensemble of ten independently trained models (named eCRRNN) is constructed. The outputs of the ensemble model are derived by averaging the individual predicted probabilities over the secondary structure labels (Eq. 9).

9 $$ y = argmax\left(\frac{1}{N}\sum\limits_{i=1}^{N}{p_{i}}\right) \\  $$

*p*_*i*_ is the output probability of constituent model and the model has been trained independently. Ensemble methods can obtain better predictive performance that could be obtained from any of the constituent predictor independently [39]. Prediction of eCRRNN achieved 74%, 76.3%, 73.9%, and 70.7% Q8 accuracy on the CB513, CASP10, CASP11, and CASP12 datasets, respectively. The Q8 prediction performance is improved by 2.6%, 2.5% 2.3% and 2% on CB513, CASP10, CASP11 and CASP12 respectively. We conducted analysis on the performance for the individual labels in CB513. Predictive accuracies of H type, E type and L type have been improved by 0.9%, 3.6% and 3.9% respectively. The secondary structures are imbalanced data and the majority labels are H, E and L. The ensemble model has effectively improved the classification accuracy for the major categories.

The precision and recall performance on the CB513 dataset are list in Table 7, and the F1 score, macro_F1, and micro_F1 are compared in Table 5. The F1 score for individual secondary structure prediction using our ensemble model was better than that of a NCCNN ensemble model. The predictive details on the CASP10, CASP11, and CASP12 datasets are listed in Table 8. We also validated its generalization on Q3 prediction and achieved 87.3%, 87.8%, 85.9% and 83.7% on CB513, CASP10, CASP11, and CASP12. Both of the Q8 and Q3 prediction results are better than the state-of-the-art.

**Table 7:** Q8 prediction using the ensemble model on the CB513 dataset

Q8 Label	precision				recall			
	eCRRNN*	CRRNN	NCCNN^a^	DCRNN^a^	eCRRNN^a^	CRRNN	NCCNN^a^	DCRNN^a^
H	**0.872**	0.860	0.846	0.832	0.935	0.926	**0.936**	0.933
B	0.582	0.466	**0.786**	0.554	0.078	**0.081**	0.047	0.026
E	**0.804**	0.797	0.776	0.753	**0.867**	0.831	0.837	0.828
G	**0.554**	0.466	0.528	0.429	**0.398**	0.371	0.29	0.252
I	0	0	0	0	0	0	0	0
T	**0.603**	0.556	**0.591**	0.559	**0.586**	0.555	0.542	0.522
S	0.563	0.494	**0.621**	0.518	**0.352**	0.332	0.237	0.249
L	**0.626**	0.603	0.570	0.573	0.697	0.658	**0.707**	0.652

^a^indicates ensemble model

Boldface numbers indicate best performance

**Table 8:** Details of Q8 accuracy on the CASP10, CASP11, and CASP12 datasets predicted by an ensemble model of CRRNN

Q8 Label	CASP10		CASP11		CASP12	
	precision	recall	precision	recall	precision	recall
H	0.894	0.925	0.867	0.931	0.853	0.926
B	0.758	0.110	0.607	0.077	0.333	0.027
E	0.829	0.868	0.796	0.864	0.746	0.837
G	0.580	0.403	0.541	0.313	0.389	0.278
I	0	0	0	0	0	0
T	0.672	0.670	0.596	0.588	0.547	0.508
S	0.561	0.366	0.523	0.327	0.490	0.263
L	0.639	0.722	0.616	0.658	0.578	0.615

The *P*-value of significance test between CRRNN and MUFOLD-SS is 5.31E-7 (< 0.005); The *P*-value of difference between eCRRNN and MUFOLD-SS is 6.93E-15; and the significance test between CRRNN and eCRRNN is at the 0.0047 level.

**Fig. 3:**
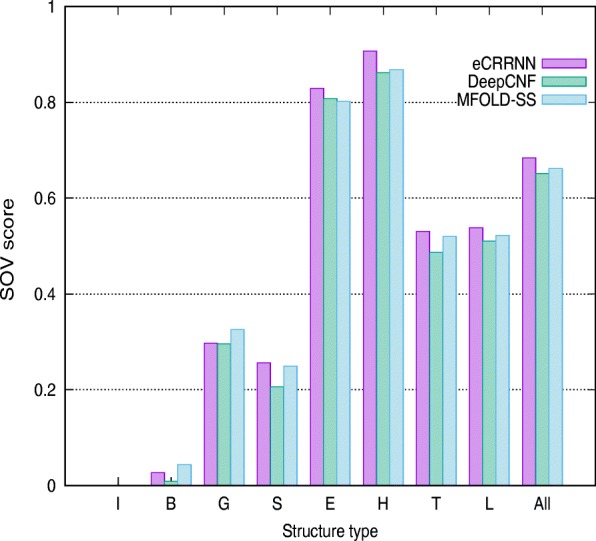
The SOV score comparison of Q8 prediction on CASP12 dataset using eCRRNN, DeepCNF and MFOLD-SS. I, B, G, S, E, H, H, T, L represent the prediction SOV score on a individual secondary structure type respectively. “All” represents the SOV score on CASP12 dataset

Segment of OVerlap(SOV) score has been used to evaluate the predicted protein secondary structures comparing with the native secondary structures. If the predictive structure segments match more native structures, SOV score will more higher. We calculate the SOV’99 score [40] using the SOV _refine [41] tool which measures how well the native and the predicted structure segments match. As shown in Table 9, in terms of SOV score on CB513, CASP10, CASP11 and CASP12, eCRRNN obtained 72.5%, 74.7%, 72.2% and 68.4% respectively. SOV scores on constituent secondary structure are also listed in Table 9. The comparison of SOV scores on CASP12 using eCRRNN, DeepCNF and MFOLD-SS is shown in Fig. 3. On the structure types B and G, the performance of eCRRNN is slightly weaker than that of MFOLD-SS. In a large number of continuous secondary structures, the performance of eCRRNN is better. Table 10 lists the detailed scores on Q3 prediction. We also compared predictive SOV score on CASP12 with JPRED, DeepCNF and MFOLD-SS, and the specific scores are listed in Table 11. Although the overall SOV score of our method is just 0.9% better than DeepCNF, the SOV score on structure C by our method is 74.1%, 8.3% better than DeepCNF. These SOV scores indicate that our method can match more continuous segments.

**Table 9:** SOV’99 scores of Q8 prediction using eCRRNN on 4 datasets: CB513, CASP10, CASP11 and CASP12

Type	CB513	CASP10	CASP11	CASP12
SOV _*L*_	0.611	0.629	0.595	0.538
SOV _*H*_	0.929	0.924	0.908	0.907
SOV _*T*_	0.599	0.67	0.605	0.531
SOV _*E*_	0.882	0.884	0.86	0.829
SOV _*S*_	0.351	0.362	0.327	0.256
SOV _*B*_	0.078	0.11	0.077	0.027
SOV _*G*_	0.419	0.416	0.339	0.297
SOV _*I*_	0	0	0	0
SOV	0.725	0.747	0.722	0.684
SOV _*mean*_	0.723	0.740	0.738	0.698

SOV _*L*_,SOV _*H*_, SOV _*T*_, SOV _*E*_, SOV _*S*_, SOV _*B*_, SOV _*G*_ and SOV _*I*_ represent the prediction SOV score on a constituent secondary structure type L, H, T, E, S,B, G and I respectively. SOV represents the SOV score on different dataset and SOV _*mean*_ is mean value of the SOV score on sequence level

**Table 10:** SOV’99 scores of Q3 prediction using eCRRNN on 4 datasets: CB513, CASP10, CASP11 and CASP12

Type	CB513	CASP10	CASP11	CASP12
SOV _*H*_	0.917	0.919	0.922	0.884
SOV _*E*_	0.859	0.868	0.835	0.798
SOV _*C*_	0.769	0.813	0.778	0.741
SOV	0.829	0.855	0.833	0.797
SOV _*mean*_	0.842	0.851	0.850	0.817

SOV _*H*_, SOV _*E*_ and SOV _*C*_ represent the prediction SOV score on a constituent secondary structure type H, E and C respectively. SOV represents the SOV score on different dataset and SOV _*mean*_ is mean value of the SOV score on sequence level

**Table 11:** SOV’99 scores of Q3 prediction on CASP12 using recently predicting methods are compared

Method	SOV _*H*_	SOV _*E*_	SOV _*C*_	SOV
JPRED	0.827	0.747	0.676	0.737
DeepCNF	0.873	0.799	0.658	0.788
MFOLD-SS	0.879	**0.814**	0.594	0.715
eCRRNN	**0.884**	0.798	**0.741**	**0.797**

SOV _*H*_, SOV _*E*_ and SOV _*C*_ represent the prediction SOV score on a constituent secondary structure type H, E and C respectively. SOV represents the SOV score on CASP12 dataset

Boldface numbers indicate best performance

Port 5 [16] is the latest release of one of the best performing secondary structure predictor. The sequences of more than 40% of the similarity with Port 5 training dataset were removed, then the four public datasets are used as validating benchmark. The Q8 prediction accuracy using Port 5 is 74%, 76.3%, 74.2%, and 70.9% respectively on CB513, CASP10, CASP11 and CASP12. The Q8 prediction accuracy using eCRRNN is 74.2%, 76.5%, 73.8%, and 70%. The SOV score measured on Port 5 is 71.3%, 73.9%, 71.8% and 67.9%. The SOV score measured on eCRRNN is 72.9%, 74.9%, 72.6% and 67.6%. Although the prediction accuracy of Port 5 on casp12 is higher than our method, it is almost the same with respect to the SOV score. The other SOV scores on our method are all better than those of Port 5. These results show that eCRRNN could obtain more meaningful secondary structure predictions.

**Fig. 4:**
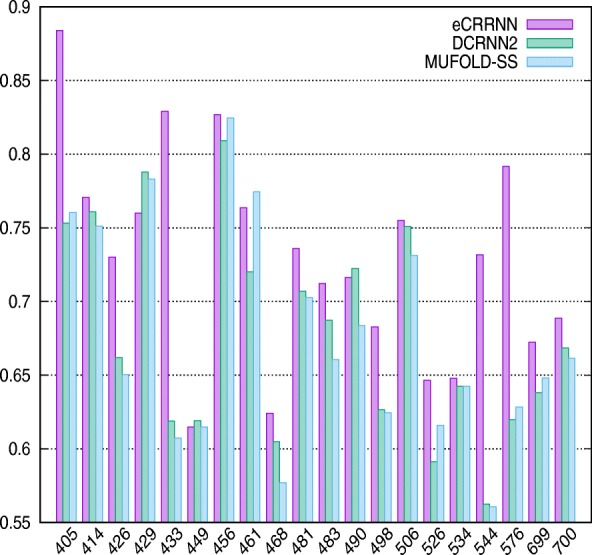
The prediction comparison of 20 sequences from CB513 dataset which sequence length is more than 400AA

Specifically, proteins of length ≥ 400AA in the CB513 dataset were 20.The performance of MUFOLD-SS and DCRNN2 is 67.12%, 67.34%. Our ensemble model achieved 72.49% accuracy on these proteins, which demonstrate the model effectiveness on capturing long-range information. The detailed performance is compared on Fig. 4.

**Fig. 5:**
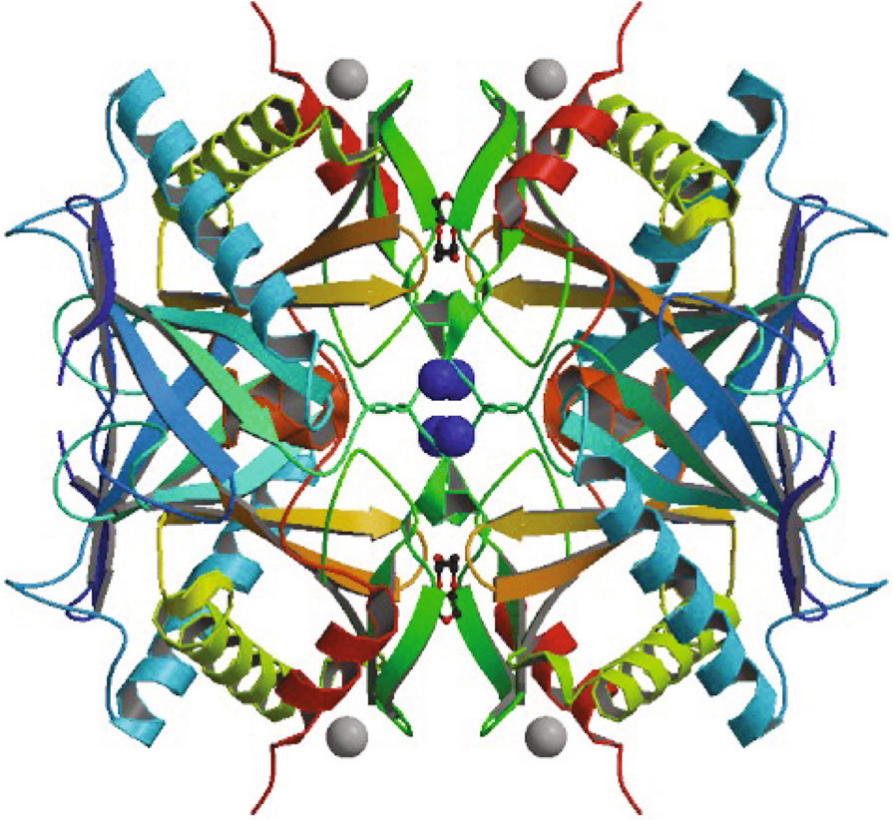
Protein 4QVU native 3D structure

**Fig. 6:**
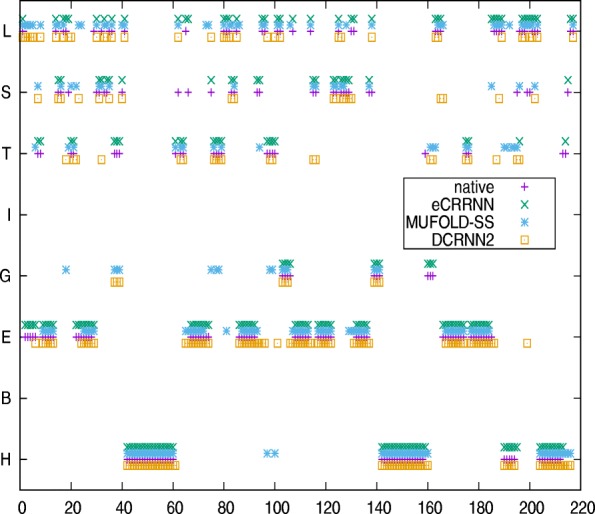
Secondary structure comparison between native and predicted structures on protein 4QVU

Two examples are used to illustrate our model performance, with the predicted results from an ensemble CRRNN(eCRRNN) model, DCRNN2 and MUFOLD-SS. A protein, T0786 (PDB-ID 4QVU), selected from the CASP11 dataset has 264 residues. The known secondary structure residues total only 217 AA (from residue 37 to 253). The native 3D structure is described in Fig. 5. The predictive accuracy according to DCRNN2, MUFOLD-SS and eCRRNN was 72.4%, 68.2%, and 91.7%. The comparison between native structure and predicted structure is described in Fig. 6. The results suggested that our model sufficiently captured continuous structure information.

**Fig. 7:**
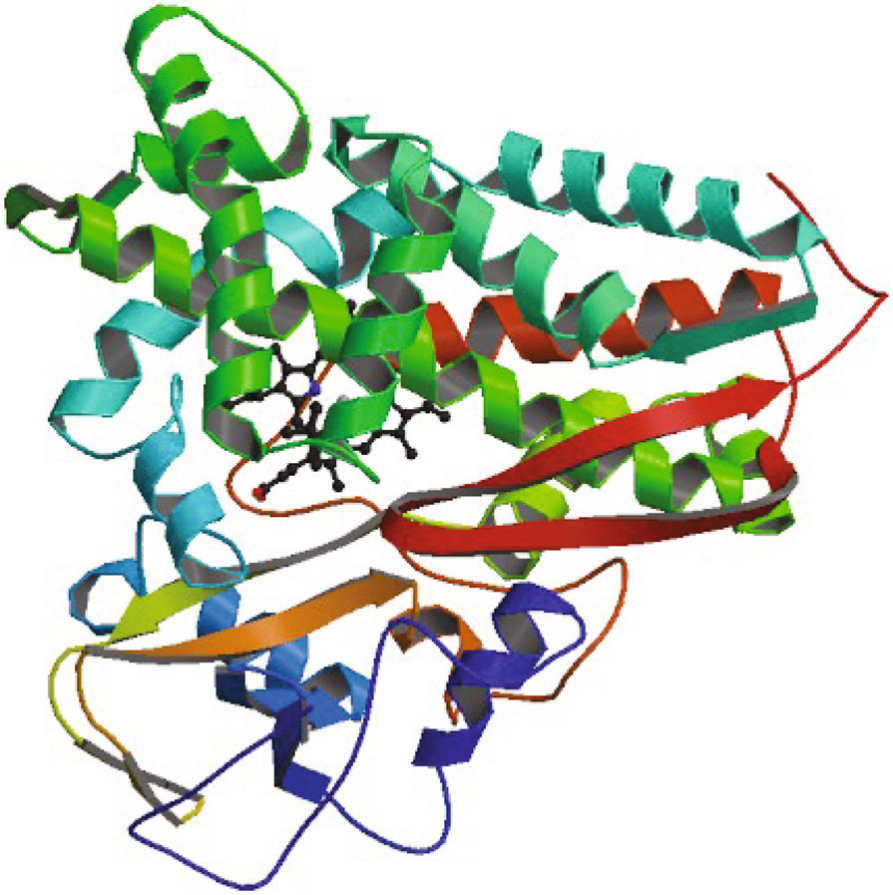
Protein 6CPP native 3D structure

**Fig. 8:**
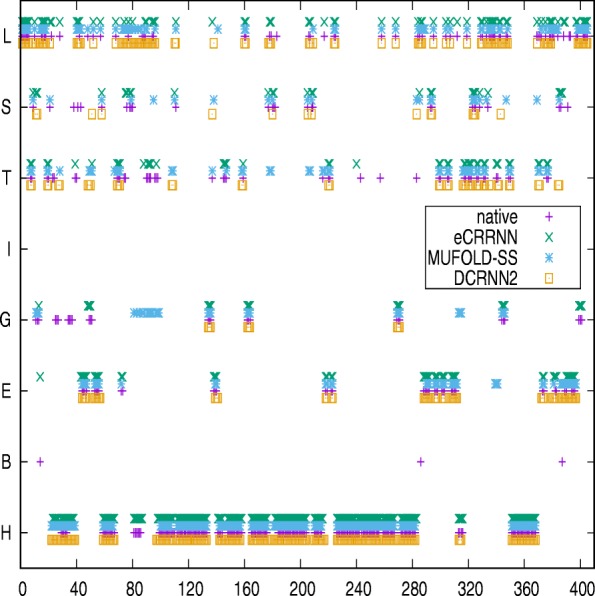
Secondary structure comparison between native and predicted structure on protein 6CPP

The 3D structure of another protein (PDB: 6CPP) selected from the CB513 dataset is shown in Fig. 7 and represents an oxidoreductase of 414 residues (only 405 residues with known structures). Predictive accuracy by DCRNN2, MUFOLD-SS, and eCRRNN was 75.3%, 76%, and 88.4%, respectively. Detailed prediction results are shown in Fig. 8. The accuracy of maximum continuous predicted structure from eCRRNN is 83AA. These results also indicate that our model was effective for long-chain protein structures. From the two cases, isolated residues which are not as same as previous and backward residue were not properly predicted, for the captured information is strongly depended on context residues.

### Ablation learning

The total parameters of our model were about 7.74 million. The feature values provided by TR5534 with 50-dimensional features were 58.777 million and the ratio of training features to model parameters was 7.6:1. The ratio of features on TR12148 to the model parameters was about 16.4:1, which is bigger than the practical requirement (10:1).

**Fig. 9:**
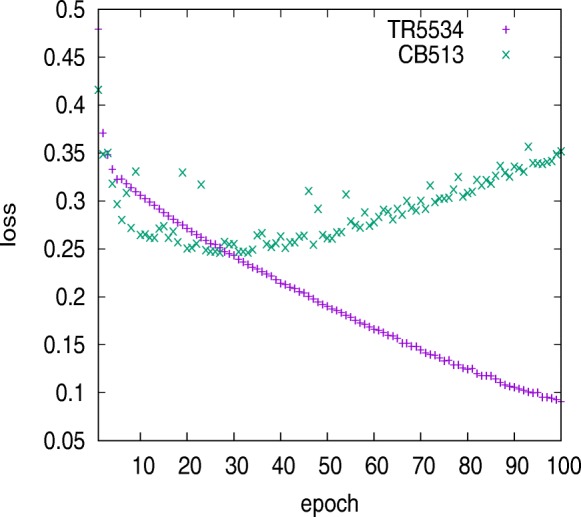
Model loss variation trained using TR5534. About 55 epochs, loss on CB513 dataset stopped decreasing and the model became overfitting

**Fig. 10:**
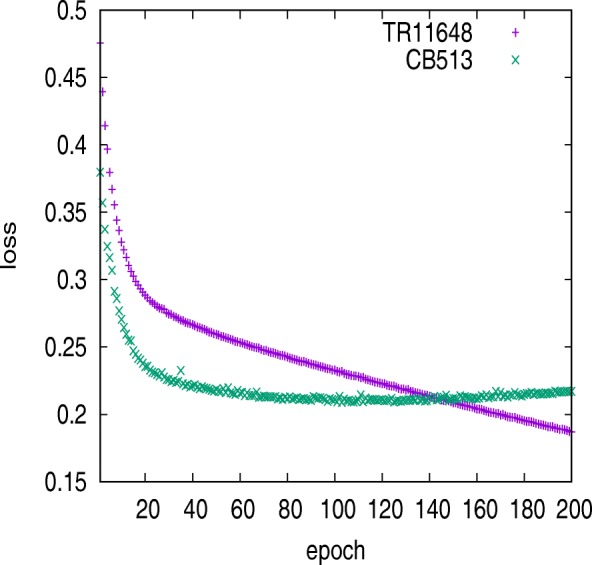
Model loss variation trained using TR12148. Loss on CB513 dataset stopped decreasing after 130 iterations and the model inclined to be stable

We trained the model using the TR5534 dataset. After about 55 epochs, the predictive accuracy for CB513 dataset decreased and the loss became increasing. The model encountered overfitting problem as Fig. 9 illustrated. The model with two BGRU layers, which were capable of reducing about 1 million parameters, was also trained using TR5534. And the prediction for CB513 dataset shows that model’s generalization was decreased. Table 12 lists the predictive performance on CB513 when model was trained using TR5534, TR6614 and TR12148. Figure 10 shows the model loss variation trained using TR12148. The training error increased along with increases in the size of the training set, because larger datasets are harder to fit. Meanwhile, the loss error of CB513 dataset was decreased, for fewer incorrect hypotheses were consistent with the training data.

**Table 12:** Model-performance comparison using different training sets against CB513

Training set	Model	Accuracy(%)
TR5534	CRRNN with 3-layer BGRU	69.6
TR5534	CRRNN with 2-layer BGRU	69.0
TR6614	CRRNN with 3-layer BGRU	70.6
TR12148	CRRNN with 3-layer BGRU	71.4

To discover important factors related to the optimal utilization of our proposed model, we evaluated alternative architectures by removing individual components. We specifically tested the performance of models without a local block or residual connections, as well as the models with 2-layer BGRUs where the input vectors were 42-dimensional features.

The test results on CB513 (Table 13) show that input features were slightly affected, and that the most important constituent was the BRNN. When input features comprised a 20-dimensional PSSM and 22-dimensional protein coding, the performance just decreased by 0.1%. When the recurrent neural network was constructed by unidirectional GRU, the performance dropped to 67.2%. Protein structure is particularly depended upon context residues; therefore, the unidirectional GRU network was ineffective at capturing contextual dependencies. Regarding the number of stacked BGRU layers, the performance of the network architecture with 1-layer was poor. When the staked layers were increased to two layers, the performance increased to 70.5%, and three-layer networks increased further to 71.4% accuracy. Increases in the stacked

**Table 13:** Comparison of different model’s generalization performance

Model	Accuracy(%)
CRRNN	71.4 ±0.2
Without ResNet	70.7 ±0.2
3-layer with BLSTM	70.2 ±0.2
Without local bolck	71.1 ±0.3
Without 1D one kernel CNN filter	71.5 ±0.2
With 2-layer BGRU	70.5 ±0.1
Unidirectional GRU	67.2
With 1-layer BGRU	69.5
CRRNN with 42dim features input	71.3 ±0.2

BRNN layers allowed the capture of more long-range information. Furthermore, the use of residual network indicated that shortcut connections between BRNN layers were essential for improving BRNN generalization. Without the residual network, accuracy dropped to 70.7%. These results are not presented on a model scale. Upon replacement of the BRNN hidden node with a LSTM, the model parameters increased to 9.99 million while the accuracy dropped to 70.2%, because the model had become overfitted and had not been adequately trained. When the 1D CNN filter with one kernel was removed, performance improved slightly improved, but 1.73 million parameters increased. These results indicated that the 1D CNN with 1 kernel effectively controlled model dimensionality without reducing model generalization. And the local block improved also overall accuracy.

## Conclusion

The CNN was successful at feature extraction, and the RNN was successful at sequence processing. Given that the residual network ImageNet [25] stacked 152 layers of convolutional neural network, we proposed a novel sequence-to-sequence deep learning model (CRRNN) for protein secondary structure prediction. Here, 1D CNN and original data were constructed into a local block to capture adjacent amino acid information. The residual network connected the interval BGRU network to improve modeling long-range dependencies. Our ensemble model was more generalizable, and the overall performance exceeded the performance by the state-of-the-art methods for both 8- and 3-state prediction. The model can also be used to predict other sequence-labeling problems and is not limited to biological problems.

## Additional file

Additional file 1 The file lists 6614 protein sequences PDB-ID which were used training in our work. (DOCX 38 kb)

## Abbreviations

BGRU: Bidirectional gated recurrent unit
BLSTM: Bidirectional long short-term memory
BRNN: Bidirectional recurrent neural network
CNN: Convolutional neural network
CRRNN: Convolutional, residual, and recurrent neural network
eCRRNN: Ensemble of convolutional, residual, and recurrent neural network
GRU: Gated recurrent units
LSTM: Long short-term memory
NCCNN: Next-step conditioned convolutional neural network
PDB: Protein data bank
PSSM: Position-specific scoring matrix
RNN: Recurrent neural network
