# Supplementary material for: Prediction of 8-state protein secondary structures by a novel deep learning architecture
Source: BMC Bioinformatics. 2018 Aug 3;19:293. doi: 10.1186/s12859-018-2280-5 (PMC6090794; doi:10.1186/s12859-018-2280-5)
Supplement: Supplementary file 1 — The file lists 6614 protein sequences PDB-ID which were used training in our work. (DOCX 38 kb) [file 12859_2018_2280_MOESM1_ESM.docx]

12ASA 16VPA 1A0EA 1A0IA 1A12A 1A5TA 1AEPA 1AIHA 1ALUA 1AM7A 1AU7A 1B33N 1B35A 1B35B 1B3TA 1B3UA 1B5LA 1B5QA 1B89A 1B8KA 1BCOA 1BCPB 1BCPD 1BCPF 1BG1A 1BG6A 1BGVA 1BHEA 1BJAA 1BOOA 1BW0A 1C0AA 1C0PA 1C1DA 1C4OA 1C8BA 1C9BA 1C9KA 1CF7B 1CFZA 1CG2A 1CJAA 1CKMA 1CNT1 1COLA 1CR1A 1CSHA 1CVJA 1CWVA 1CZNA 1D6MA 1D7MA 1DABA 1DBHA 1DJ7A 1DJ7B 1DKGA 1DMLA 1DMUA 1DPGA 1E3JA 1E44B 1E94E 1E9RA 1EBFA 1EBPA 1EDZA 1EFYA 1EI5A 1EI7A 1EI9A 1EK9A 1ELUA 1EPUA 1ESCA 1ETEA 1EV7A 1EVSA 1EZ0A 1F0LA 1F20A 1F35A 1F5QB 1F60A 1F7UA 1F89A 1F8MA 1FC6A 1FCDA 1FG7A 1FIOA 1FJRA 1FLOA 1FOEA 1FXKA 1G31A 1G8MA 1G8PA 1GD8A 1GNWA 1GP6A 1GPJA 1GTTA 1GU3A 1GVEA 1GXCA 1GXSB 1GZSB 1H12A 1H21A 1H2BA 1H30A 1H3GA 1H5WA 1H6DA 1H80A 1H9AA 1HCNB 1HCUA 1HDHA 1HDRA 1HEKA 1HF2A 1HJ6A 1HKQA 1HLMA 1HO8A 1HR6B 1HRUA 1HSTA 1HULA 1HX8A 1HYHA 1HYNP 1I1QA 1I1RB 1I2KA 1I3JA 1I4DA 1I4WA 1I58A 1I5PA 1I8NA 1I9ZA 1IARB 1ID1A 1IE7C 1IGNA 1IGWA 1IJ5A 1IM3D 1IRXA 1ITBB 1ITHA 1IXHA 1IXMA 1IZCA 1IZOA 1J4AA 1J5YA 1J6RA 1JB0D 1JB0E 1JB0F 1JB0L 1JB7A 1JDCA 1JEYA 1JEYB 1JFIA 1JG5A 1JK0B 1JL5A 1JLYA 1JOFA 1JQLB 1JS8A 1JSSA 1JTVA 1JU2A 1JX7A 1JYEA 1K0IA 1K78A 1K8QA 1K8TA 1KA8A 1KCFA 1KCGC 1KDGA 1KF6C 1KHXA 1KI9A 1KJNA 1KMIZ 1KNGA 1KNXA 1KNYA 1KO7A 1KOLA 1KQ3A 1KTGA 1KVKA 1KWAA 1KXPD 1KXUA 1KYQA 1L0SA 1L1OC 1L8WA 1LBQA 1LEHA 1LF6A 1LFBA 1LFDA 1LFWA 1LJ2A 1LJ8A 1LRZA 1LW7A 1M0UA 1M1EB 1M1ZA 1M32A 1M3YA 1M4IA 1M4UA 1M6SA 1M6YB 1MDAH 1MIJA 1MIWA 1MJNA 1MKFA 1MNAA 1MO9A 1MSPA 1MXRA 1N13B 1N4KA 1N7VA 1NA6A 1NBWA 1NBWB 1NG0A 1NGMB 1NH1A 1NHYA 1NI5A 1NIJA 1NQKA 1NXHA 1NXUA 1O59A 1O65A 1O69A 1O70A 1O7DE 1O88A 1O98A 1O9YA 1OAPA 1ODHA 1OF5B 1OFUX 1OH2Q 1OJ5A 1OKCA 1OKGA 1OLTA 1OMZA 1OR4A 1OYGA 1OZ2A 1OZJA 1P32A 1P3RA 1P4CA 1P9LA 1P9YA 1PAQA 1PBYB 1PC3A 1PC6A 1PEAA 1PIXA 1PJHA 1POIA 1POIB 1PPJB 1PPJD 1PXUA 1PXZA 1PYAA 1PYAB 1Q06A 1Q15A 1Q1HA 1Q67A 1Q74A 1Q8CA 1Q9JA 1QCXA 1QD6C 1QEXA 1QFJA 1QHDA 1QI9A 1QKSA 1QQRA 1QSMA 1QTFA 1QYIA 1QYSA 1QZZA 1R0MA 1R2JA 1R71A 1R89A 1R8GA 1R8IA 1RCWA 1RDO1 1RF6A 1RI6A 1RJDA 1RL6A 1RLZA 1RMGA 1RO2A 1RSGA 1RTWA 1RYIA 1RYPL 1S0PA 1S48A 1S98A 1SAZA 1SCFA 1SDDA 1SEFA 1SEZA 1SHUX 1SL8A 1SO7A 1SP3A 1SQ5A 1SQHA 1SRQA 1STZA 1SVSA 1SW6A 1SZIA 1SZQA 1T08A 1T0IA 1T4OA 1T4WA 1T77A 1T8SA 1TD6A 1TFZA 1THTA 1TO3A 1TTZA 1TULA 1TV8A 1TVFA 1TWFF 1TXGA 1TXKA 1U0JA 1U0MA 1U19A 1U2CA 1U2KA 1U2MA 1U2ZA 1U7ZA 1U8SA 1U8XX 1UA7A 1UHVA 1UJWB 1UNQA 1UPSA 1UQWA 1US5A 1USUB 1UTYA 1UURA 1UX5A 1UYNX 1V72A 1V9DA 1VA6A 1VBVA 1VF7A 1VG0A 1VI1A 1VJGA 1VJQA 1VJVA 1VL4A 1VP7A 1VPQA 1VQOB 1VQOL 1VQOM 1VQON 1VQOP 1VQOQ 1VQOT 1VQOW 1VQOX 1VQOZ 1VRBA 1VSGA 1W1WE 1W33A 1W4XA 1W5TA 1W9YA 1W9ZA 1WBAA 1WF3A 1WLEA 1WM1A 1WMIA 1WOUA 1WPXB 1WTHD 1WWJA 1WY5A 1WY6A 1WY9A 1X3LA 1X6MA 1X9ZA 1XAWA 1XDIA 1XDYA 1XECA 1XG8A 1XIPA 1XJUA 1XK5A 1XKPC 1XKRA 1XKSA 1XKZA 1XL3C 1XLQA 1XLYA 1XMTA 1XMXA 1XNXA 1XO0A 1XO1A 1XODA 1XOUA 1XOUB 1XOVA 1XPKA 1XPPA 1XQAA 1XQOA 1XR4A 1XRSA 1XSVA 1XSZA 1XTPA 1XTTA 1XV5A 1XWVA 1Y08A 1Y0KA 1Y0NA 1Y12A 1Y14A 1Y1PA 1Y28A 1Y3TA 1Y43B 1Y5HA 1Y60A 1Y6XA 1Y6ZA 1Y71A 1Y8AA 1Y8XB 1Y96A 1Y9BA 1Y9IA 1YA0A 1YA5T 1YACA 1YARO 1YAVA 1YC9A 1YD0A 1YD7A 1YDXA 1YDYA 1YF2A 1YF3A 1YFQA 1YGAA 1YGTA 1YHNB 1YHTA 1YI9A 1YISA 1YK3A 1YKDA 1YKHB 1YKIA 1YLIA 1YLLA 1YLMA 1YLOA 1YLXA 1YN3A 1YNFA 1YNPA 1YNRA 1YOVA 1YOZA 1YP0A 1YPXA 1YPYA 1YQGA 1YQSA 1YQTA 1YQZA 1YREA 1YRRA 1YRTA 1YS1X 1YT3A 1YT8A 1YU0A 1YUEA 1YUMA 1YVWA 1YW4A 1YX1A 1YY7A 1YZYA 1Z0NA 1Z0PA 1Z1YA 1Z2NX 1Z2WA 1Z3EA 1Z4RA 1Z67A 1Z6RA 1Z70X 1Z72A 1Z7MA 1Z84A 1Z94A 1ZA7A 1ZB1A 1ZBOA 1ZBXB 1ZCEA 1ZEEA 1ZGKA 1ZH8A 1ZHVA 1ZHXA 1ZI8A 1ZJCA 1ZK4A 1ZK5A 1ZK8A 1ZL0A 1ZLDA 1ZMTA 1ZS4A 1ZSQA 1ZT3A 1ZTDA 1ZTHA 1ZUNA 1ZVAA 1ZVPA 1ZWWA 1ZXKA 1ZXMA 1ZY7A 1ZY9A 1ZYLA 1ZYNA 1ZYQA 1ZZ1A 1ZZKA 2A06B 2A1HA 2A1JA 2A1KA 2A1RA 2A1VA 2A1XA 2A2FX 2A2MA 2A35A 2A3NA 2A4XA 2A5HA 2A5YB 2A65A 2A6HE 2A6SA 2A6ZA 2A9IA 2AAMA 2ABSA 2ADVC 2AEBA 2AEGA 2AEUA 2AG4A 2AGKA 2AHDA 2AHMA 2AHUA 2AJ7A 2AJAA 2AJRA 2AKZA 2AL6A 2AMHA 2AMYA 2ANEA 2ANUA 2AO9A 2AP3A 2APJA 2APLA 2AQ4A 2AQ6A 2AQWA 2ARCA 2ASBA 2ASTB 2ATZA 2AU5A 2AVDA 2AVWA 2AWIA 2AXQA 2AZ0A 2AZ4A 2AZEA 2AZEB 2B0VA 2B1EA 2B1YA 2B2AA 2B4JC 2B4VA 2B4WA 2B61A 2B69A 2B78A 2B81A 2B82A 2B8IA 2B99A 2B9WA 2BA2A 2BASA 2BB6A 2BBDA 2BBRA 2BE1A 2BF6A 2BFCA 2BFDA 2BFDB 2BFEA 2BGCA 2BGHA 2BH1X 2BHVA 2BHWA 2BIBA 2BIFA 2BIIA 2BIVA 2BJ0A 2BJFA 2BJNA 2BJQA 2BKFA 2BKWA 2BKXA 2BLLA 2BLNA 2BM8A 2BMOA 2BMOB 2BNLA 2BO4A 2BOLA 2BONA 2BOUA 2BPA1 2BPSA 2BRYA 2BS2C 2BSJA 2BT9A 2BU3A 2BVFA 2BWFA 2BWRA 2BZ1A 2BZ4A 2BZVA 2C0CA 2C0GA 2C0NA 2C1DA 2C1LA 2C1VA 2C1WA 2C2IA 2C2QA 2C3VA 2C4JA 2C5AA 2C5KT 2C5LC 2C5RA 2C5UA 2C5WB 2C61A 2C78A 2C8MA 2C9WA 2CA6A 2CASA 2CB2A 2CBZA 2CCMA 2CCVA 2CDCA 2CDUA 2CFMA 2CFQA 2CH5A 2CH7A 2CHOA 2CI1A 2CIBA 2CKKA 2CLYB 2CMGA 2CMZA 2CNQA 2CO5A 2COVD 2CVIA 2CW6A 2CW9A 2CWRA 2CWYA 2CX7A 2CXAA 2CXHA 2CXIA 2CXNA 2CXYA 2CZLA 2CZVC 2D00A 2D0BA 2D0TA 2D1GA 2D1SA 2D2SA 2D3DA 2D42A 2D4PA 2D4XA 2D54A 2D5BA 2D5FA 2D5MA 2D5WA 2D68A 2D74B 2D7EA 2D7IA 2D7VA 2D80A 2D81A 2D9RA 2DBNA 2DBSA 2DBYA 2DCLA 2DDRA 2DDUA 2DDXA 2DDZA 2DE3A 2DE6A 2DEJA 2DF7A 2DG1A 2DGDA 2DGKA 2DH2A 2DI4A 2DJFA 2DKAA 2DKJA 2DKOB 2DLAA 2DLBA 2DOQD 2DPFA 2DPLA 2DPMA 2DQLA 2DRUA 2DS2B 2DSKA 2DSLA 2DT8A 2DTJA 2DU3A 2DULA 2DVMA 2DWKA 2DXAA 2DXUA 2DY0A 2DYIA 2DYTA 2E11A 2E1FA 2E1MA 2E1MB 2E1VA 2E2OA 2E3HA 2E4MC 2E4TA 2E52A 2E56A 2E5FA 2E5YA 2E6FA 2E6MA 2E7JA 2E8BA 2E8EA 2E8VA 2E9XD 2ECEA 2ED6A 2EFJA 2EFKA 2EFVA 2EGVA 2EHPA 2EHZA 2EI9A 2EIYA 2EK0A 2EKDA 2EKLA 2ELCA 2ENDA 2EPLX 2ERFA 2ERVA 2ES4D 2ETJA 2EV1A 2EWTA 2EX2A 2EX5A 2EY4C 2EZ2A 2F1FA 2F22A 2F23A 2F2CA 2F31A 2F48A 2F4NA 2F5TX 2F5UA 2F62A 2F6HX 2F6KA 2F6MB 2F7BA 2F7VA 2F8LA 2F9FA 2F9IA 2F9IB 2FA1A 2FA8A 2FAOA 2FB0A 2FB5A 2FBAA 2FBIA 2FCAA 2FCJA 2FCKA 2FCLA 2FCTA 2FCWA 2FCWB 2FDOA 2FEFA 2FELA 2FEPA 2FEXA 2FF4A 2FFGA 2FFMA 2FFSA 2FGGA 2FGQX 2FGTA 2FGYA 2FHDA 2FHPA 2FHZA 2FHZB 2FI9A 2FIQA 2FIUA 2FIYA 2FJ8A 2FJI1 2FK6A 2FKCA 2FKKA 2FLIA 2FM9A 2FMYA 2FNAA 2FNJB 2FNUA 2FOZA 2FP4B 2FP8A 2FPHX 2FPNA 2FQ3A 2FQXA 2FR5A 2FREA 2FSJA 2FSQA 2FTWA 2FU2A 2FULA 2FURA 2FWHA 2FY7A 2FYGA 2FZSA 2G0DA 2G30A 2G3RA 2G3VA 2G3WA 2G45A 2G5GX 2G6TA 2G7SA 2G8SA 2G9WA 2GA1A 2GA8A 2GAGB 2GAGC 2GAKA 2GB3A 2GB4A 2GB7A 2GBLA 2GBOA 2GD5A 2GDQA 2GEXA 2GF3A 2GF4A 2GGOA 2GHSA 2GHTA 2GIAA 2GIBA 2GIYA 2GJ2A 2GJLA 2GJXA 2GK9A 2GKEA 2GKGA 2GKPA 2GLFA 2GMFA 2GMHA 2GMQA 2GNOA 2GNXA 2GPIA 2GPJA 2GQWA 2GR8A 2GRCA 2GREA 2GRRB 2GRVA 2GSCA 2GSOA 2GT1A 2GTIA 2GU3A 2GUDA 2GUFA 2GUIA 2GUKA 2GUZB 2GV5C 2GWDA 2GWFA 2GWGA 2GWMA 2GYQA 2GYSA 2GZ6A 2GZAA 2H1CA 2H1TA 2H1VA 2H21A 2H5NA 2H62C 2H7OA 2H7ZA 2H88A 2H88B 2H88C 2H88D 2H8GA 2H9AA 2H9AB 2HA9A 2HALA 2HAZA 2HB0A 2HBOA 2HBVA 2HCFA 2HD9A 2HDWA 2HEUA 2HEWF 2HFSA 2HFTA 2HHCA 2HHZA 2HIMA 2HINA 2HIQA 2HIVA 2HJ1A 2HJEA 2HJMA 2HKJA 2HKVA 2HL0A 2HLJA 2HLRA 2HLYA 2HLZA 2HMAA 2HNGA 2HNUA 2HO0A 2HOXA 2HP0A 2HQ7A 2HQBA 2HQLA 2HQSA 2HQTA 2HQYA 2HRAA 2HS1A 2HSBA 2HUEB 2HUEC 2HUHA 2HW2A 2HWJA 2HWKA 2HX0A 2HX5A 2HXTA 2HY5A 2HY5C 2HY7A 2HYPA 2HYTA 2HZCA 2HZLA 2HZMA 2HZMB 2I00A 2I06A 2I0KA 2I0MA 2I0ZA 2I15A 2I1SA 2I2CA 2I2LA 2I2XA 2I3OA 2I44A 2I49A 2I4LA 2I53A 2I5HA 2I5IA 2I5VO 2I6HA 2I6TA 2I71A 2I74A 2I7GA 2I7HA 2I7RA 2I7XA 2I8DA 2I9CA 2I9IA 2I9WA 2IA1A 2IA7A 2IAFA 2IAYA 2IC6A 2ICHA 2ICSA 2ICWG 2ICYA 2ID4A 2IF6A 2IFUA 2IG3A 2IGPA 2IGSA 2IGTA 2IH2A 2IHTA 2II0A 2II2A 2IIDA 2IIHA 2IJ2A 2IJLA 2IJRA 2IKSA 2IL5A 2ILKA 2IM8A 2IMFA 2IMHA 2IMJA 2IMQX 2IMRA 2IMSA 2IN3A 2IN5A 2INUA 2IP1A 2IP2A 2IP6A 2IQIA 2IT9A 2ITBA 2IUUA 2IUYA 2IVFC 2IVNA 2IW1A 2IWRA 2IXAA 2IXMA 2IXSA 2IY2A 2IYFA 2IYKA 2IYVA 2IZ6A 2IZRA 2IZWA 2J16A 2J1DG 2J1VA 2J3TC 2J43A 2J4DA 2J4OA 2J58A 2J5BA 2J66A 2J6AA 2J6LA 2J73A 2J7QA 2J8AA 2J8BA 2J8GA 2J91A 2J97A 2J9OA 2J9WA 2JA9A 2JAEA 2JBWA 2JC9A 2JD3A 2JDCA 2JDIA 2JDID 2JDIH 2JDJA 2JE3A 2JEEA 2JEKA 2JFKA 2JFRA 2JG0A 2JG1A 2JGPA 2JH3A 2JHPA 2JIIA 2JISA 2JJQA 2JKGA 2JLIA 2JLNA 2JLQA 2LISA 2MBRA 2MCMA 2MEV1 2MPRA 2NLVA 2NMLA 2NMMA 2NN4A 2NNUA 2NOOA 2NPSD 2NPTA 2NQ2A 2NQTA 2NRHA 2NRJA 2NRQA 2NRRA 2NS0A 2NS6A 2NSAA 2NSMA 2NT0A 2NTPA 2NTXA 2NVOA 2NW8A 2NWAA 2NWFA 2NWHA 2NWLA 2NXFA 2NXOA 2NXPA 2NXVA 2NXWA 2NYKA 2NZ7A 2NZCA 2NZLA 2NZXA 2O0JA 2O0MA 2O0QA 2O0TA 2O14A 2O2KA 2O2XA 2O30A 2O34A 2O38A 2O3AA 2O3IA 2O3JA 2O3OA 2O4AA 2O4TA 2O4VA 2O5NA 2O5VA 2O62A 2O6KA 2O6PA 2O70A 2O7GA 2O7RA 2O8PA 2O8SA 2O90A 2O9SA 2OA5A 2OB3A 2OB9A 2OBDA 2OBNA 2OBPA 2OD4A 2OD5A 2ODAA 2ODFA 2ODIA 2ODVA 2OEBA 2OEEA 2OEXA 2OEZA 2OF3A 2OFCA 2OFKA 2OG4A 2OGBA 2OGFA 2OGGA 2OH3A 2OHWA 2OITA 2OIZA 2OIZD 2OJWA 2OKTA 2OKUA 2OLNA 2OLTA 2OMLA 2OMZA 2ONDA 2OOIA 2OPCA 2OPEA 2OQBA 2ORWA 2ORYA 2OSXA 2OTMA 2OU1A 2OU3A 2OU5A 2OU6A 2OV0A 2OWLA 2OX6A 2OX7A 2OXLA 2OXOA 2OY9A 2OYOA 2OYYA 2OYZA 2OZ8A 2OZGA 2OZJA 2OZLA 2OZNB 2OZTA 2P02A 2P0AA 2P0LA 2P0NA 2P0OA 2P0SA 2P0WA 2P12A 2P14A 2P17A 2P1MB 2P22C 2P26A 2P2SA 2P38A 2P3EA 2P3PA 2P3XA 2P3YA 2P4GA 2P4HX 2P4OA 2P51A 2P53A 2P58C 2P5ZX 2P62A 2P65A 2P67A 2P6VA 2P6WA 2P8GA 2P8IA 2P9BA 2P9WA 2P9XA 2PA7A 2PAGA 2PBEA 2PCSA 2PD0A 2PD2A 2PEFA 2PFTA 2PFZA 2PGCA 2PGEA 2PGNA 2PGSA 2PH5A 2PH7A 2PHNA 2PHPA 2PIAA 2PIFA 2PIGA 2PIHA 2PJDA 2PJPA 2PKEA 2PKFA 2PKHA 2PM7A 2PMAA 2PMLX 2PMVA 2PN1A 2PNEA 2PNLA 2PNQA 2PNWA 2POCA 2POFA 2POMA 2PORA 2PP6A 2PPQA 2PQ7A 2PQ8A 2PRSA 2PRVA 2PRXA 2PS1A 2PS5A 2PSBA 2PSPA 2PSTX 2PT7G 2PTRA 2PUZA 2PV2A 2PV4A 2PW0A 2PWWA 2PXXA 2PY5A 2PY6A 2PYGA 2PYQA 2PYWA 2PYXA 2Q00A 2Q01A 2Q03A 2Q04A 2Q07A 2Q0SA 2Q0XA 2Q0ZX 2Q12A 2Q1WA 2Q22A 2Q28A 2Q2IA 2Q35A 2Q3SA 2Q40A 2Q43A 2Q48A 2Q4AA 2Q4OA 2Q4WA 2Q4ZA 2Q58A 2Q66A 2Q6KA 2Q6QA 2Q7AA 2Q7SA 2Q82A 2Q83A 2Q88A 2Q8KA 2Q8PA 2Q9KA 2Q9RA 2Q9UA 2QA1A 2QAEA 2QAPA 2QASA 2QB7A 2QCPX 2QCUA 2QDFA 2QDJA 2QDLA 2QDRA 2QE8A 2QEEA 2QFEA 2QG7A 2QGMA 2QGQA 2QGVA 2QGYA 2QH9A 2QHFA 2QHPA 2QHQA 2QIBA 2QIPA 2QJ8A 2QJFA 2QJLA 2QJTB 2QJVA 2QKDA 2QKHA 2QL8A 2QM8A 2QMAA 2QMLA 2QN6B 2QNGA 2QNKA 2QNLA 2QNUA 2QOLA 2QP2A 2QPXA 2QQ4A 2QQ8A 2QR4A 2QR6A 2QRLA 2QRUA 2QRYA 2QSDA 2QSFA 2QSFX 2QSIA 2QSKA 2QSVA 2QSWA 2QTQA 2QTSA 2QU7A 2QU8A 2QUFA 2QUOA 2QV3A 2QV6A 2QW5A 2QWWA 2QXFA 2QYAA 2QYFB 2QYVA 2QYWA 2QZCA 2QZUA 2R01A 2R0HA 2R0XA 2R16A 2R19A 2R1FA 2R2CA 2R2YA 2R2ZA 2R31A 2R3SA 2R41A 2R44A 2R4FA 2R4IA 2R51A 2R5OA 2R5SA 2R60A 2R6JA 2R6ZA 2R751 2R78A 2R7DA 2R7GA 2R85A 2R8WA 2R91A 2R9FA 2R9IA 2RA1A 2RA8A 2RAAA 2RAUA 2RBCA 2RCCA 2RD7A 2RDGA 2RDPA 2RDQA 2REUA 2RFFA 2RFQA 2RH0A 2RH3A 2RHMA 2RHSA 2RILA 2RINA 2RJ2A 2RJBA 2RJIA 2RJNA 2RJOA 2RJZA 2RKHA 2RKNA 2RKVA 2RL8A 2RLDA 2SAKA 2SPCA 2TNFA 2TPSA 2TRCP 2UURA 2UUZA 2UVKA 2UW1A 2UWJE 2UXEA 2UXQA 2UY1A 2UYOA 2UYTA 2UZ1A 2UZCA 2V05A 2V0HA 2V0PA 2V1MA 2V1YB 2V2GA 2V33A 2V3AA 2V3IA 2V3MA 2V3SA 2V66B 2V6GA 2V6VA 2V73A 2V75A 2V76A 2V7FA 2V7KA 2V84A 2V89A 2V8IA 2V8QA 2V8QB 2V8TA 2V9KA 2V9PA 2V9VA 2VA0A 2VAKA 2VBKA 2VBUA 2VC8A 2VCHA 2VCLA 2VDFA 2VDJA 2VDUB 2VDWA 2VDWB 2VE3A 2VE8A 2VEQA 2VEZA 2VFKA 2VFOA 2VFRA 2VFXA 2VGLB 2VGLS 2VGNA 2VH3A 2VHAA 2VHHA 2VHKA 2VK2A 2VK8A 2VLAA 2VLDA 2VLGA 2VLQA 2VLQB 2VN6A 2VNGA 2VNUD 2VOKA 2VOUA 2VOVA 2VPNA 2VPTA 2VPVA 2VPZC 2VQ2A 2VQCA 2VQGA 2VQPA 2VRSA 2VS7A 2VSGA 2VSOE 2VSYA 2VTYA 2VUNA 2VVMA 2VVPA 2VW8A 2VWAA 2VWSA 2VXBA 2VXGA 2VXNA 2VXPA 2VXXA 2VY1A 2W07B 2W0GA 2W15A 2W18A 2W1RA 2W2DB 2W2RA 2W2SA 2W2XD 2W31A 2W3PA 2W3QA 2W3XA 2W40A 2W42A 2W45A 2W4SA 2W4YA 2W50A 2W56A 2W5QA 2W61A 2W68A 2W6AA 2W7NA 2W7VA 2W7ZA 2W83C 2W8MA 2W8TA 2W8XA 2W91A 2W9JA 2W9YA 2WAAA 2WAOA 2WAWA 2WB0X 2WB7A 2WBIA 2WDCA 2WDQC 2WDQD 2WE5A 2WE5B 2WE8A 2WEFA 2WF7A 2WFPA 2WFWA 2WG8A 2WH6A 2WH7A 2WI8A 2WJ5A 2WJNC 2WJRA 2WK1A 2WLRA 2WLUA 2WLVA 2WNFA 2WNKA 2WNPF 2WOLA 2WOYA 2WP0C 2WP7A 2WPXA 2WQ4A 2WSDA 2WSWA 2WT7B 2WTPA 2WUQA 2WUXA 2WVQA 2WW5A 2WWXB 2WY3B 2WY8Q 2WYAA 2WYLA 2WZBA 2WZKA 2WZOA 2WZPR 2X0DA 2X12A 2X1DA 2X27X 2X29A 2X2SA 2X2UA 2X3DA 2X3GA 2X3HA 2X3JA 2X3LA 2X3MA 2X3NA 2X49A 2X4GA 2X4JA 2X4LA 2X55A 2X5CA 2X5FA 2X5HA 2X5OA 2X5QA 2X5RA 2X5XA 2X5YA 2X65A 2X6NA 2X6WA 2X7IA 2X7QA 2X8TA 2X9ZA 2XBGA 2XC8A 2XCBA 2XCIA 2XD7A 2XDGA 2XDJA 2XEDA 2XEPA 2XESA 2XETA 2XEUA 2XFGB 2XFNA 2XFRA 2XFVA 2XG5B 2XGFA 2XGRA 2XHAA 2XHFA 2XHGA 2XHIA 2XI7A 2XIOA 2XKOC 2XLTA 2XM5A 2XMIA 2XMJA 2XMOA 2XMPA 2XN6A 2XOCA 2XODA 2XOMA 2XPIA 2XPPA 2XQHA 2XQQA 2XQUA 2XQXA 2XRHA 2XRYA 2XSEA 2XSKA 2XT2A 2XTSA 2XU0A 2XU3A 2XU8A 2XUAA 2XUBA 2XUVA 2XVEA 2XVYA 2XWSA 2XWVA 2XXNA 2XXPA 2XYIA 2XYKA 2XZ9A 2XZEA 2XZIA 2Y1EA 2Y1HA 2Y24A 2Y26A 2Y44A 2Y48B 2Y4YA 2Y53A 2Y6UA 2Y7PA 2Y8DA 2Y8NB 2Y9UA 2Y9WA 2Y9WC 2YANA 2YAVA 2YC2A 2YC3A 2YCHA 2YEQA 2YEVC 2YFKA 2YFRA 2YFUA 2YGBA 2YGKA 2YGUA 2YH5A 2YH6A 2YHAA 2YHGA 2YIJA 2YIMA 2YINA 2YIZA 2YJGA 2YK4A 2YKFA 2YKZA 2YLMA 2YLNA 2YMAA 2YMOA 2YMVA 2YN0A 2YN5A 2YNAA 2YNKA 2YNMC 2YNMD 2YORA 2YQ2A 2YV4A 2YV5A 2YVTA 2YWIA 2YX0A 2YXNA 2YXYA 2YY3A 2YYKA 2YYYA 2YZSA 2YZTA 2YZVA 2YZYA 2Z07A 2Z08A 2Z0BA 2Z0DA 2Z0JA 2Z0QA 2Z0RA 2Z0TA 2Z0XA 2Z14A 2Z1DA 2Z26A 2Z2NA 2Z3QA 2Z4SA 2Z51A 2Z5BA 2Z5EA 2Z6OA 2Z6RA 2Z72A 2Z81A 2Z84A 2Z8FA 2Z8ZA 2Z9WA 2ZA4B 2ZAHA 2ZB4A 2ZB6A 2ZB9A 2ZBLA 2ZCAA 2ZCMA 2ZD7A 2ZDIC 2ZDJA 2ZDSA 2ZE7A 2ZF9A 2ZFDB 2ZFGA 2ZFYA 2ZFZA 2ZGIA 2ZGYA 2ZHJA 2ZIHA 2ZIVB 2ZK9X 2ZKOA 2ZKTA 2ZKZA 2ZMEB 2ZMEC 2ZNRA 2ZO4A 2ZOGA 2ZOSA 2ZOUA 2ZPMA 2ZQ5A 2ZQKC 2ZQOA 2ZSJA 2ZTBA 2ZU9A 2ZUXA 2ZW5A 2ZWAA 2ZWSA 2ZXEB 2ZXXC 2ZY4A 2ZYRA 2ZYZB 2ZZJA 3A07A 3A09A 3A0YA 3A16A 3A1JA 3A1JB 3A1JC 3A1PB 3A2EA 3A35A 3A57A 3A5FA 3A5PA 3A5YA 3A6FA 3A6RA 3A72A 3A77A 3A8GB 3A8RA 3A98B 3A9BA 3A9FA 3A9IA 3A9LA 3A9SA 3AA0B 3AAFA 3AB1A 3ABHA 3ABIA 3ABQA 3ABQB 3ACXA 3AEHA 3AEIA 3AFCA 3AFOA 3AG3A 3AG3B 3AG3E 3AG3F 3AG3G 3AG7A 3AGKA 3AHNA 3AIAA 3AJ1A 3AJ7A 3AJAA 3AJFA 3AJIB 3AKJA 3AKSA 3AL2A 3ALJA 3AMIA 3AMLA 3AMRA 3AOFA 3AONA 3AONB 3AOTA 3AOWA 3AP1A 3AQ2A 3AQBA 3AQEA 3AQOA 3AS8A 3ASLA 3ATSA 3AV3A 3AWMA 3AWUA 3AX1A 3AX2A 3AXBA 3AXGA 3AY5A 3AYHA 3AYHB 3B0DC 3B0XA 3B33A 3B40A 3B47A 3B49A 3B4NA 3B4QA 3B4UA 3B59A 3B5EA 3B5OA 3B5QA 3B6EA 3B6HA 3B7CA 3B7FA 3B7KA 3B8FA 3B8OA 3B8XA 3B9OA 3B9TA 3B9WA 3BA3A 3BAMA 3BB0A 3BC8A 3BC9A 3BE3A 3BEDA 3BF5A 3BF7A 3BFMA 3BFNA 3BFQG 3BG1B 3BG2A 3BH0A 3BH1A 3BH4A 3BH7B 3BHDA 3BHWA 3BIOA 3BIQA 3BIYA 3BJDA 3BJEA 3BJNA 3BJQA 3BK3C 3BL9A 3BM1A 3BM3A 3BMAA 3BMZA 3BN0A 3BN3B 3BNVA 3BO6A 3BODA 3BOEA 3BONA 3BPJA 3BPKA 3BPOC 3BPTA 3BPVA 3BQ9A 3BQAA 3BQKA 3BQOA 3BQPA 3BQWA 3BQXA 3BRCA 3BRFA 3BRKX 3BRVB 3BS0A 3BS4A 3BS6A 3BS7A 3BSOA 3BT3A 3BT4A 3BT5A 3BT7A 3BTPA 3BUUA 3BUXB 3BVOA 3BWSA 3BWUF 3BWWA 3BWZA 3BXJA 3BXWA 3BXWB 3BY7A 3BY9A 3BYPA 3BYQA 3BYWA 3BZ5A 3BZMA 3BZWA 3C02A 3C0DA 3C0FB 3C0WA 3C18A 3C1AA 3C1QA 3C1YA 3C25A 3C2GA 3C2QA 3C2UA 3C37A 3C3DA 3C3VA 3C4AA 3C4NA 3C4RA 3C5EA 3C5KA 3C5NA 3C5PA 3C5VA 3C6AA 3C6FA 3C6KA 3C70A 3C7AA 3C8CA 3C8DA 3C8GA 3C8IA 3C8LA 3C8VA 3C8WA 3C8YA 3C8ZA 3C96A 3C9FA 3C9HA 3C9UA 3CAIA 3CAWA 3CBPA 3CBWA 3CBZA 3CC1A 3CCDA 3CDDA 3CE9A 3CEBA 3CECA 3CETA 3CEUA 3CEXA 3CG6A 3CGXA 3CHBD 3CHHA 3CHJA 3CHMA 3CI0I 3CI0K 3CI3A 3CIJA 3CINA 3CISA 3CJDA 3CJLA 3CJSB 3CJXA 3CJYA 3CKCA 3CKDA 3CKJA 3CKMA 3CL5A 3CL6A 3CLJA 3CLMA 3CLQA 3CLWA 3CM1A 3CMNA 3CNBA 3CNIA 3CNYA 3COQA 3COVA 3CP0A 3CP3A 3CPQA 3CPTB 3CPXA 3CQ1A 3CQ5A 3CQCA 3CQCB 3CQXC 3CQYA 3CR3A 3CRJA 3CRKC 3CRRA 3CRVA 3CSVA 3CSXA 3CT5A 3CT6A 3CT9A 3CTPA 3CTRA 3CTWB 3CTZA 3CU9A 3CUZA 3CVEA 3CWCA 3CWFA 3CWNA 3CWRA 3CX2A 3CX4A 3CX5A 3CX5B 3CX5C 3CX5G 3CXBA 3CXJA 3CXNA 3CYMA 3CZ1A 3CZ8A 3CZPA 3D00A 3D06A 3D0FA 3D0KA 3D19A 3D1BA 3D1CA 3D1KA 3D1PA 3D1RA 3D2OA 3D32A 3D33A 3D34A 3D37A 3D3BA 3D3BJ 3D3KA 3D3YA 3D4EA 3D4UB 3D59A 3D6IA 3D6RA 3D6WA 3D7AA 3D7IA 3D7RA 3D8KA 3D8LA 3D8PA 3D8UA 3DA0A 3DA1A 3DA5A 3DA7A 3DADA 3DANA 3DB2A 3DBGA 3DBYA 3DCPA 3DCZA 3DD7A 3DDCB 3DDEA 3DDQB 3DDVA 3DEEA 3DEFA 3DELB 3DF6A 3DF7A 3DFEA 3DFFA 3DG6A 3DGPA 3DGPB 3DH4A 3DHAA 3DHUA 3DHXA 3DI2A 3DI5A 3DJEA 3DJLA 3DJMA 3DK9A 3DKXA 3DLCA 3DMBA 3DMEA 3DMGA 3DMNA 3DMYA 3DN7A 3DNHA 3DNJA 3DNSA 3DO6A 3DO9A 3DOEB 3DORA 3DOUA 3DP7A 3DPTA 3DR2A 3DR5A 3DR7A 3DRAB 3DRFA 3DS4A 3DSDA 3DSKA 3DSMA 3DSOA 3DSSA 3DT5A 3DTDA 3DTEA 3DTYA 3DTZA 3DUPA 3DUZA 3DV8A 3DVOA 3DW8B 3DWCA 3DWOX 3DXEA 3DXIA 3DXLA 3DXPA 3DXRA 3DXRB 3DYDA 3DYJA 3DYTA 3DZAA 3DZMA 3E0EA 3E0JB 3E0RA 3E0SA 3E0ZA 3E1EA 3E35A 3E38A 3E3MA 3E3XA 3E4BA 3E4GA 3E4VA 3E4WA 3E59A 3E5TA 3E61A 3E6CC 3E8LC 3E8VA 3E96A 3E99A 3E9KA 3EAFA 3EB8A 3EBBA 3EBCA 3EBEA 3EBWA 3EC3A 3ECFA 3EDPA 3EDYA 3EE4A 3EEBA 3EEHA 3EEQA 3EERA 3EF8A 3EGAA 3EGRA 3EGWC 3EHCA 3EI3B 3EIPA 3EJKA 3EKIA 3EL6A 3ELBA 3ELEA 3ELFA 3ELIA 3ELKA 3EMIA 3ENCA 3EO6A 3EO7A 3EOIA 3EOJA 3EOQA 3EPHA 3EPSA 3EQXA 3ER6A 3ER7A 3ER9B 3ESLA 3ESSA 3ETJA 3ETOA 3ETVA 3ETZA 3EUHA 3EUHC 3EURA 3EVIA 3EVNA 3EVYA 3EWDA 3EXZA 3EZ0A 3EZ2A 3EZIA 3F0DA 3F0HA 3F1IH 3F1PB 3F1TA 3F2ZA 3F3KA 3F40A 3F4LA 3F4MA 3F62A 3F6VA 3F75P 3F79A 3F7EA 3F85A 3F8DA 3F8TA 3F95A 3F9SA 3FAKA 3FAYA 3FBGA 3FBQA 3FBZA 3FCGA 3FCNA 3FD5A 3FD9A 3FDEA 3FDHA 3FDSC 3FEFA 3FEGA 3FETA 3FF1A 3FFRA 3FFVA 3FFYA 3FGAB 3FGRA 3FGRB 3FGVA 3FGXA 3FH3A 3FHDA 3FHHA 3FHLA 3FI9A 3FIAA 3FIDA 3FJ1A 3FJUB 3FJYA 3FK4A 3FK8A 3FKAA 3FKCA 3FKEA 3FKJA 3FKQA 3FKRA 3FLOA 3FLOB 3FM0A 3FM5A 3FMCA 3FN5A 3FNBA 3FNCA 3FNDA 3FNIA 3FO3A 3FOKA 3FOTA 3FOVA 3FP3A 3FPCA 3FPPA 3FQ8A 3FRHA 3FRNA 3FS3A 3FSOA 3FSSA 3FSTA 3FT1A 3FTJA 3FUCA 3FUTA 3FV9A 3FVZA 3FW9A 3FWKA 3FWYA 3FWZA 3FX3A 3FX7A 3FXBA 3FYMA 3FZ2A 3G02A 3G0TA 3G16A 3G1JA 3G1PA 3G21A 3G23A 3G2EA 3G3LA 3G3SA 3G3TA 3G40A 3G46A 3G4NA 3G5BA 3G5SA 3G5TA 3G6EY 3G7PA 3G7QA 3G7UA 3G85A 3G8QA 3G8RA 3G8YA 3G91A 3G9HA 3G9MA 3GA4A 3GA7A 3GAEA 3GB5A 3GBGA 3GBVA 3GBWA 3GD0A 3GD6A 3GD7A 3GDWA 3GE3A 3GE3B 3GE3C 3GE3E 3GETA 3GFAA 3GFFA 3GG7A 3GGNA 3GGYA 3GHAA 3GHMA 3GIAA 3GIUA 3GIWA 3GJYA 3GK7A 3GKEA 3GKJA 3GKMA 3GKRA 3GMGA 3GMIA 3GMXA 3GN3A 3GN6A 3GNEA 3GNFB 3GO2A 3GO9A 3GOCA 3GODA 3GOEA 3GOHA 3GONA 3GOZA 3GP4A 3GP6A 3GPVA 3GQHA 3GQVA 3GQXA 3GR3A 3GR4A 3GR5A 3GRAA 3GREA 3GRHA 3GRLA 3GS9A 3GSEA 3GTZA 3GUDA 3GV0A 3GVAA 3GVEA 3GVOA 3GVZA 3GW4A 3GWBA 3GWLA 3GWQA 3GWRA 3GXHA 3GYCA 3GZAA 3GZBA 3GZRA 3H05A 3H0DA 3H0UA 3H14A 3H16A 3H1DA 3H1TA 3H20A 3H2GA 3H2YA 3H2ZA 3H35A 3H38A 3H3LA 3H49A 3H4CA 3H4LA 3H4TA 3H4XA 3H5LA 3H5TA 3H63A 3H6EA 3H6JA 3H6QA 3H6RA 3H75A 3H79A 3H7CX 3H7HB 3H7IA 3H7LA 3H8GA 3H8GF 3H9CA 3H9MA 3H9PA 3HBCA 3HBXA 3HBZA 3HC1A 3HCYA 3HDJA 3HDXA 3HFHA 3HFIA 3HFOA 3HFWA 3HGTA 3HGUA 3HH1A 3HHWA 3HHWK 3HI0A 3HIDA 3HIEA 3HJ4A 3HJHA 3HKAA 3HKLA 3HKWA 3HL1A 3HL2A 3HL6A 3HLKA 3HLSA 3HLUA 3HLXA 3HLZA 3HMSA 3HN0A 3HN2A 3HN7A 3HNOA 3HOIA 3HOLA 3HPCX 3HPFA 3HPYA 3HQ1A 3HQFA 3HR0A 3HR6A 3HRDC 3HRGA 3HRLA 3HRPA 3HRQA 3HRZA 3HSAA 3HSIA 3HSLX 3HSUA 3HSYA 3HTKB 3HTSB 3HTUA 3HTYA 3HUFA 3HUGB 3HUTA 3HUUA 3HV8A 3HVAA 3HWUA 3HWWA 3HX3A 3HX9A 3HXJA 3HXLA 3HY0A 3HYIA 3HYNA 3HYWA 3HZ6A 3HZ8A 3HZPA 3HZSA 3I00A 3I09A 3I0PA 3I0ZA 3I10A 3I16A 3I18A 3I1AA 3I2DA 3I2KA 3I2NA 3I33A 3I3VA 3I3WA 3I45A 3I4GA 3I4OA 3I4ZA 3I53A 3I57A 3I5QA 3I5TA 3I6EA 3I6XA 3I76A 3I7AA 3I7MA 3I83A 3I84A 3I8BA 3I94A 3I9YA 3IABA 3IACA 3IAGC 3IARA 3IB5A 3IB7A 3IBWA 3IC8A 3IC9A 3ID1A 3IDBB 3IDFA 3IDWA 3IE4A 3IEGA 3IEKA 3IEYB 3IFEA 3IFRA 3IFUA 3IG2A 3IG5A 3IG9A 3IGFA 3IGHX 3IGQA 3IGZB 3IH6A 3IHMA 3IHVA 3II2A 3IIBA 3IIIA 3IISM 3IIXA 3IJ6A 3IJDA 3IJLA 3IJMA 3IJWA 3IK7A 3IKWA 3ILVA 3ILWA 3IM1A 3IM6A 3INGA 3IO1A 3IO3A 3IO5A 3IOHA 3IOXA 3IP0A 3IP3A 3IP4B 3IPJA 3IQ0A 3IQWA 3IRBA 3IRPX 3IRVA 3IS6A 3ISMA 3ISMC 3ISQA 3ISYA 3IT3A 3IT4A 3IT4B 3IT5A 3IT8D 3ITEA 3ITFA 3ITQA 3IU0A 3IU6A 3IUKA 3IUOA 3IUPA 3IUSA 3IUUA 3IUVA 3IUWA 3IUZA 3IV0A 3IV3A 3IV7A 3IVEA 3IVVA 3IWFA 3IX0A 3IX3A 3IXLA 3IXSA 3JQ0A 3JQ1A 3JQOA 3JR1A 3JRNA 3JRVA 3JS6A 3JS8A 3JSRA 3JSYA 3JSZA 3JTMA 3JTNA 3JTXA 3JTZA 3JU2A 3JU7A 3JUMA 3JURA 3JV1A 3JVOA 3JX9A 3JXPA 3JXYA 3JY6A 3JYBA 3JYOA 3JZLA 3JZYA 3K01A 3K05A 3K0ZA 3K11A 3K13A 3K1HA 3K1TA 3K1UA 3K26A 3K29A 3K2IA 3K2OA 3K2YA 3K2ZA 3K3CA 3K3FA 3K3VA 3K40A 3K4IA 3K4TA 3K5JA 3K63A 3K69A 3K6MA 3K6QA 3K6YA 3K7CA 3K7IB 3K85A 3K8PC 3K8PD 3K8RA 3K94A 3K9TA 3KA7A 3KAEA 3KB2A 3KB9A 3KBGA 3KBRA 3KC2A 3KD3A 3KD4A 3KD6A 3KDGA 3KDRA 3KE3A 3KEPA 3KEYA 3KF6B 3KF8B 3KFFA 3KFOA 3KG7A 3KG9A 3KGKA 3KGWA 3KH1A 3KH8A 3KHKA 3KIZA 3KJXA 3KK7A 3KKGA 3KKIA 3KLJA 3KLQA 3KLUA 3KM5A 3KMIA 3KNYA 3KNZA 3KOGA 3KORA 3KP1E 3KP8A 3KQ0A 3KQ5A 3KQNA 3KS3A 3KS6A 3KS9A 3KSNA 3KSPA 3KSXA 3KT7A 3KTCA 3KTDA 3KTOA 3KUVA 3KVCA 3KVHA 3KW2A 3KWEA 3KWLA 3KWRA 3KXEA 3KXPA 3KXRA 3KXSA 3KXWA 3KXYA 3KXYT 3KY8A 3KYAA 3KYJA 3KYLA 3KYZA 3KZHA 3KZJA 3L00A 3L09A 3L0AA 3L0QA 3L15A 3L1LA 3L1WA 3L22A 3L2CA 3L39A 3L4CA 3L4FA 3L51B 3L5AA 3L60A 3L6BA 3L6IA 3L6TA 3L7HA 3L81A 3L82B 3L8WA 3LAGA 3LAXA 3LB2A 3LC0A 3LD1A 3LD7A 3LDCA 3LDQB 3LDUA 3LEDA 3LEQA 3LETA 3LEWA 3LFTA 3LFUA 3LGBA 3LGDA 3LH2S 3LHCA 3LHIA 3LHNA 3LHOA 3LHXA 3LICA 3LIUA 3LJBA 3LJNA 3LKBA 3LKDA 3LKEA 3LKMA 3LL3A 3LL7A 3LLKA 3LLPA 3LM2A 3LM3A 3LM4A 3LMAA 3LMBA 3LMFA 3LMMA 3LNBA 3LNLA 3LNNA 3LO8A 3LOGA 3LOPA 3LOVA 3LP5A 3LPHA 3LPZA 3LQ9A 3LQBA 3LQKA 3LRQA 3LRVA 3LS9A 3LSNA 3LSOA 3LTIA 3LURA 3LUTA 3LUYA 3LVUA 3LWTX 3LWXA 3LXQA 3LXRF 3LXZA 3LY1A 3LY7A 3LYBA 3LYEA 3LYGA 3LYHA 3LYSA 3LYWA 3LZDA 3LZKA 3M03A 3M07A 3M0MA 3M1CB 3M1TA 3M1UA 3M1XA 3M2PA 3M2TA 3M31A 3M33A 3M3PA 3M4IA 3M5QA 3M6IA 3M6JA 3M6NA 3M6ZA 3M73A 3M7AA 3M7KA 3M7NA 3M7OA 3M84A 3M89A 3M8JA 3M9VA 3MABA 3MADA 3MAHA 3MBRX 3MC3A 3MC9A 3MCPA 3MCRA 3MCWA 3MCXA 3MCZA 3MDMA 3MDNA 3MDQA 3MDUA 3ME0B 3MEAA 3MEMA 3MFBA 3MFIA 3MFXA 3MGAA 3MGBA 3MHXA 3MI01 3MILA 3MIZA 3MJ0A 3MJEA 3MJFA 3MJGA 3MJOA 3MK4A 3MKCA 3MKHA 3MKOA 3MKRB 3MKZA 3MLNA 3MLQA 3MMHA 3MMPF 3MMPG 3MMYA 3MOIA 3MP7A 3MPKA 3MQ0A 3MQ1A 3MQDA 3MR0A 3MRUA 3MSOA 3MSQA 3MSWA 3MSXB 3MT0A 3MT1A 3MT5A 3MTSA 3MTVA 3MTWA 3MV2B 3MVAO 3MVCA 3MVNA 3MW8A 3MWCA 3MWDA 3MWXA 3MWZA 3MXNA 3MXNB 3MXOA 3MXZA 3MY2A 3MYDA 3MYOA 3MZ0A 3MZ2A 3MZKB 3N01A 3N08A 3N0AA 3N0WA 3N0XA 3N17A 3N1EA 3N1MC 3N29A 3N2WA 3N2ZB 3N3MA 3N40P 3N4SA 3N54B 3N6TA 3N6XA 3N6YA 3N6ZA 3N7XA 3N89A 3N8BA 3N91A 3N9YA 3NA5A 3NBCA 3NBIA 3NBXX 3NC3A 3NCEA 3NCTA 3ND1A 3NE5B 3NE8A 3NEHA 3NEKA 3NETA 3NFGA 3NFGB 3NFTA 3NG7X 3NG9A 3NGQA 3NGXA 3NHEA 3NI0A 3NIXA 3NJDA 3NJEA 3NKEA 3NKSA 3NKUA 3NLCA 3NNBA 3NNFA 3NO0A 3NO2A 3NOQA 3NPDA 3NPHB 3NPPA 3NQIA 3NQNA 3NQZA 3NR5A 3NRAA 3NREA 3NRFA 3NRHA 3NRLA 3NRSA 3NRWA 3NRXA 3NS4A 3NTXA 3NUFA 3NUQA 3NV0A 3NVNB 3NVOA 3NVSA 3NVWB 3NVXA 3NW4A 3NWRA 3NX4A 3NY3A 3NYBA 3NYMA 3NYTA 3NYWA 3NZLA 3NZTA 3O0GD 3O0QA 3O0YA 3O0ZA 3O10A 3O12A 3O1IC 3O2TA 3O3MA 3O3MB 3O4HA 3O4PA 3O53A 3O5YA 3O65A 3O6QA 3O6ZA 3O7BA 3O7IA 3O83A 3O8MA 3O8QA 3O9ZA 3OA5A 3OAEA 3OAJA 3OAMA 3OAOA 3OB6A 3OBEA 3OBLA 3OC8A 3OCJA 3OCUA 3OD1A 3OD8A 3ODMA 3ODNA 3ODTA 3OE3A 3OEPA 3OF4A 3OF6D 3OFGA 3OG6B 3OGNA 3OH8A 3OHEA 3OHGA 3OHSX 3OIQA 3OIYA 3OKGA 3OKPA 3OKQA 3OKXA 3OKZA 3OL2B 3OLCX 3OM0A 3OMBA 3ON5A 3ONDA 3OO8A 3OOQA 3OOSA 3OOXA 3OP6A 3OQ4A 3OQBA 3OQQA 3OR1A 3OR1B 3ORJA 3ORKA 3ORUA 3OS4A 3OSEA 3OSTA 3OSVA 3OT1A 3OT2A 3OT9A 3OTDA 3OTIA 3OTNA 3OUNB 3OUVA 3OV9A 3OVBA 3OVGA 3OW8A 3OWRA 3OY2A 3OYVA 3OYZA 3OZ2A 3OZPA 3P02A 3P0BA 3P0KA 3P0UA 3P0WA 3P1VA 3P24A 3P2CA 3P2EA 3P2HA 3P2MA 3P2TA 3P3DA 3P3YA 3P4GA 3P51A 3P5JA 3P5JB 3P6LA 3P7IA 3P8AA 3P8CF 3P9VA 3P9ZA 3PB6X 3PBPA 3PBTA 3PC7A 3PDYA 3PE5A 3PE6A 3PE7A 3PE9A 3PESA 3PEVB 3PF0A 3PF2A 3PF7A 3PFEA 3PFGA 3PFOA 3PG6A 3PG7A 3PGUA 3PH9A 3PHXA 3PHXB 3PICA 3PIJA 3PIUA 3PJ0A 3PJPA 3PKOA 3PKZA 3PL0A 3PL2A 3PM9A 3PMCA 3PMEA 3PMGA 3PMOA 3PMSA 3PN3A 3PNNA 3PNRB 3PNXA 3POHA 3POJA 3POPA 3POWA 3PP2A 3PP5A 3PPLA 3PPMA 3PR6A 3PROC 3PS0A 3PSHA 3PT1A 3PT5A 3PT8B 3PTYA 3PU2A 3PU5A 3PU9A 3PUCA 3PUIA 3PV8A 3PVCA 3PVEA 3PVHA 3PVIA 3PVKA 3PVTA 3PVZA 3PW3A 3PXLA 3PYCA 3PYWA 3PZ6A 3PZDA 3PZSA 3Q0HA 3Q18A 3Q1CA 3Q1IA 3Q1XA 3Q20A 3Q2IA 3Q2UA 3Q3MB 3Q3QA 3Q46A 3Q49B 3Q6AA 3Q6BA 3Q6CA 3Q6KA 3Q7CA 3Q7RA 3Q8DA 3QBTB 3QC5X 3QC7A 3QD7X 3QDKA 3QE7A 3QE9Y 3QECA 3QEEA 3QEKA 3QELB 3QF2A 3QF7A 3QFEA 3QFTA 3QFWA 3QGUA 3QH6A 3QH9A 3QHBA 3QHEA 3QHOA 3QHQA 3QI7A 3QJ4A 3QL6A 3QL9A 3QM9A 3QMLC 3QNSA 3QOWA 3QP4A 3QP9A 3QQ2A 3QQ5A 3QQYA 3QQZA 3QR7A 3QRAA 3QS2A 3QSGA 3QSJA 3QSLA 3QSQA 3QSZA 3QT2A 3QTAA 3QTHA 3QTMA 3QU3A 3QU5A 3QUFA 3QVLA 3QVPA 3QVSA 3QWBA 3QWEA 3QWGA 3QWLA 3QWMA 3QWNA 3QWUA 3QXBA 3QXFA 3QXZA 3QY3A 3QY7A 3QYEA 3QYFA 3QZMA 3QZXA 3R07C 3R15A 3R1KA 3R24A 3R2CA 3R2QA 3R41A 3R44A 3R45C 3R4IA 3R4VA 3R4ZA 3R5TA 3R6DA 3R6UA 3R72A 3R84A 3R8JA 3R9FA 3RAMA 3RAUA 3RBYA 3RC1A 3RC9A 3RCOA 3RD7A 3RENA 3RF3A 3RFAA 3RFEA 3RFYA 3RG8A 3RGCA 3RGQA 3RHZA 3RIMA 3RJ2X 3RJOA 3RJPA 3RJUA 3RJVA 3RK1A 3RK6A 3RKCA 3RKGA 3RKLA 3RKOB 3RKOC 3RKOD 3RKOG 3RKOM 3RKON 3RL5A 3RLFF 3RLFG 3RLKA 3RLOA 3RLSA 3RMQA 3RNLA 3RNVA 3ROBA 3RONA 3RPCA 3RPDA 3RPFA 3RPJA 3RPPA 3RQ1A 3RQAA 3RQTA 3RQWA 3RRIA 3RRKA 3RSNA 3RT3C 3RTLA 3RUIA 3RVAA 3RX6A 3RX9A 3RXYA 3RY3A 3RZAA 3RZIA 3RZNA 3S0AA 3S21A 3S25A 3S2JA 3S2KC 3S2RA 3S44A 3S5JB 3S5WA 3S63A 3S64A 3S6PA 3S79A 3S8GA 3S8IA 3S8MA 3S8SA 3S98A 3S99A 3S9JA 3SAMA 3SB4A 3SBMA 3SBTB 3SC0A 3SC7X 3SCYA 3SDBA 3SEOA 3SFVB 3SG0A 3SGGA 3SGHA 3SGWA 3SHGA 3SHGB 3SHPA 3SHQA 3SHSA 3SIGA 3SJ5A 3SJAC 3SK7A 3SK9A 3SKQA 3SKVA 3SL9A 3SLRA 3SMPA 3SMTA 3SNKA 3SNOA 3SO6A 3SOJA 3SOKA 3SONA 3SOYA 3SPEA 3SQ7A 3SQLA 3SQZA 3SREA 3SRIA 3SS7X 3SSOA 3STOA 3SU6A 3SWHA 3SWOA 3SX6A 3SXOA 3SY1A 3SYLA 3SZ3A 3SZAA 3SZVA 3SZYA 3T0HA 3T2CA 3T2LA 3T33A 3T3LA 3T3OA 3T4LA 3T5NA 3T5VA 3T5VB 3T5XA 3T61A 3T6AA 3T6GB 3T6OA 3T6SA 3T7AA 3T7DA 3T7KA 3T7ZA 3T8JA 3T8KA 3T92A 3T9OA 3TACB 3TAIA 3TBDA 3TC3A 3TC8A 3TCMA 3TCVA 3TD7A 3TDGA 3TDQA 3TDWA 3TE6A 3TE8A 3TEBA 3TEJA 3TEKA 3TEUA 3TFGA 3TFJA 3TG9A 3THIA 3TIPA 3TIXB 3TJ1A 3TJMA 3TJZB 3TJZC 3TK8A 3TL8B 3TLQA 3TM4A 3TM8A 3TMGA 3TNYA 3TOVA 3TPDA 3TQQA 3TRKA 3TS3A 3TS9A 3TSAA 3TT2A 3TT9A 3TTGA 3TU8A 3TUFA 3TUIA 3TUOA 3TUTA 3TVJA 3TVKA 3TVQA 3TW5A 3TW8A 3TWDA 3TWLA 3TX8A 3TXNA 3TXSA 3TXVA 3TZYA 3U02A 3U07A 3U0HA 3U0RA 3U0VA 3U12A 3U1UA 3U21A 3U24A 3U2UA 3U3LC 3U4GA 3U4KA 3U4VA 3U4YA 3U4ZA 3U52A 3U52C 3U5VA 3U5WA 3U64A 3U65A 3U65B 3U6XS 3U7QA 3U7QB 3U7ZA 3U81A 3U8VA 3U97A 3U9JA 3U9RB 3U9WA 3UA0A 3UAFA 3UANA 3UAUA 3UB1A 3UB2A 3UBYA 3UCSA 3UD1A 3UEBA 3UEJA 3UF7A 3UFBA 3UFIA 3UG9A 3UGFA 3UGOA 3UGUA 3UIDA 3UIFA 3UITA 3ULBA 3ULJA 3UMHA 3UMOA 3UMZA 3UN6A 3UOAB 3UP6A 3UPLA 3UPSA 3UR8A 3URZA 3US3A 3USHA 3UT4A 3UUEA 3UUNA 3UUWA 3UV1A 3UW3A 3UWPA 3UWSA 3UWSB 3UXFA 3V0DA 3V0RA 3V1VA 3V2UA 3V33A 3V39A 3V3LA 3V3NA 3V3TA 3V42A 3V46A 3V47C 3V4CA 3V5CA 3V5RA 3V5UA 3V69A 3V6IA 3V6OA 3V71A 3V75A 3V7DB 3V7NA 3V85A 3V93A 3V96A 3V9OA 3V9WA 3VA9A 3VBCA 3VC1A 3VC8A 3VCAA 3VDJA 3VENA 3VG8A 3VGIA 3VGLA 3VGPA 3VGZA 3VHJA 3VHLA 3VHXB 3VIQA 3VIQB 3VJ9A 3VJFA 3VJJA 3VK5A 3VKWA 3VL1A 3VL9A 3VLAA 3VLDA 3VMNA 3VN3A 3VNRA 3VNYA 3VOQA 3VORA 3VOTA 3VP9A 3VPBA 3VPZA 3VQFA 3VQJA 3VQTA 3VR0A 3VR4A 3VRDB 3VRHA 3VS8A 3VSJB 3VSVA 3VU1A 3VU4A 3VU9B 3VUBA 3VUEA 3VUPA 3VV1A 3VVVA 3VWAA 3VWBA 3VWNX 3VX0A 3VX3A 3VXCA 3VXJA 3VXVA 3VY8X 3VYWA 3VZ6A 3VZ9B 3VZHA 3VZXA 3W06A 3W07A 3W0FA 3W0KA 3W0OA 3W0RA 3W15A 3W15B 3W1EA 3W20A 3W2WA 3W2WB 3W36A 3W3WA 3W42A 3W4TA 3W54A 3W5HA 3W5SA 3W6JA 3W6KB 3W6SA 3W8QA 3W9EA 3W9SA 3WA1A 3WA2X 3WARA 3WASA 3WCOA 3WCTB 3WDCA 3WDGB 3WDNA 3WDQA 3WE0A 3WECA 3WFDB 3WG9A 3WGQA 3WGTA 3WGXA 3WH2A 3WHRA 3WHXB 3WI3A 3WI5A 3WISA 3WIWA 3WJ9A 3WJDA 3WJPA 3WKGA 3WKRC 3WKXA 3WKYA 3WL4A 3WL8A 3WMTA 3WMVA 3WMWA 3WNDA 3WNZA 3WO6A 3WOEB 3WOLA 3WPUA 3WQCA 3WQMA 3WQTA 3WRBA 3WRYA 3WSXA 3WT0A 3WTDA 3WTTB 3WU2A 3WU2B 3WU2H 3WU2O 3WURA 3WV4A 3WVQA 3WW9A 3WWCA 3WX4A 3WX7A 3WXFA 3WXMB 3WXYA 3WZSA 3X01B 3X0UA 3X0VA 3X1LB 3X1LC 3X1LH 3X27A 3X3EA 3ZBGA 3ZBOA 3ZC0A 3ZC4A 3ZCOA 3ZDOA 3ZDSA 3ZEUB 3ZF8A 3ZFPA 3ZGJA 3ZH9B 3ZHEA 3ZHEB 3ZHIA 3ZIAA 3ZIDA 3ZIEA 3ZIGA 3ZIHA 3ZILA 3ZJ0A 3ZJAA 3ZJBA 3ZJEA 3ZK4A 3ZL8A 3ZLCA 3ZN3A 3ZN4A 3ZN6A 3ZNVA 3ZOJA 3ZPJA 3ZPLA 3ZPNA 3ZPXA 3ZQUA 3ZR8X 3ZRGA 3ZRXA 3ZSUA 3ZTAA 3ZTHA 3ZTVA 3ZUIA 3ZUZA 3ZVLA 3ZWFA 3ZX3A 3ZX7A 3ZXCA 3ZXKA 3ZXNA 3ZY2A 3ZY7A 3ZYPA 3ZYTA 3ZZHA 3ZZOA 4A02A 4A0EA 4A1GA 4A1RA 4A20A 4A27A 4A29A 4A2BA 4A2VA 4A35A 4A37A 4A3PA 4A56A 4A5UA 4A5UB 4A69C 4A6DA 4A6QA 4A7UA 4A8JA 4A8JB 4A8JC 4A9AA 4A9CA 4A9VA 4AB5A 4ABMA 4ABYA 4ACFA 4ACOA 4ACVA 4ADIA 4ADMA 4ADNA 4ADZA 4AE0A 4AE7A 4AEQA 4AEZA 4AF1A 4AF8A 4AFFA 4AFKA 4AG6A 4AGKA 4AGSA 4AJSA 4AJYC 4AJYV 4AKFA 4AKKA 4AKLA 4AKMA 4AKXA 4AL0A 4ALZA 4AMQA 4ANNA 4ANOA 4AP5A 4AQ4A 4AQLA 4AQNA 4AQOA 4AR9A 4ARTA 4ARUA 4AS2A 4ASCA 4ASMB 4AT0A 4AT7A 4AT7B 4ATGA 4ATHA 4ATMA 4AU1A 4AUKA 4AURA 4AVRA 4AVSA 4AW7A 4AXDA 4AXOA 4AY7A 4AY9A 4AYOA 4AZ6A 4AZSA 4B0MA 4B0TA 4B1MA 4B1YB 4B21A 4B28A 4B2FA 4B46A 4B4DA 4B4UA 4B4YA 4B5OA 4B60A 4B6HA 4B87A 4B89A 4B8EA 4B8VA 4B8XA 4B91A 4B9GA 4BAXA 4BB9A 4BC3A 4BE3A 4BESA 4BEUA 4BFCA 4BFOA 4BG2A 4BG7A 4BGBA 4BGCA 4BGPA 4BH6A 4BHQA 4BHRA 4BHUA 4BJ0A 4BJ1A 4BJIA 4BJJB 4BJMA 4BJQA 4BJUA 4BJZA 4BK0A 4BK7A 4BKWA 4BL6A 4BLPA 4BLQA 4BLUA 4BMJA 4BN4A 4BNDA 4BOEA 4BOJA 4BOUA 4BPFA 4BPSA 4BPUB 4BPZA 4BQ6D 4BQHA 4BQNA 4BQQA 4BQYA 4BRCA 4BSPA 4BSVA 4BSXA 4BT7A 4BT9A 4BUGA 4BUOA 4BVNA 4BVQA 4BWDA 4BWRA 4BWZA 4BX9C 4BXFA 4BXOB 4BY6A 4BYZA 4BZ4A 4BZAA 4BZPA 4C08A 4C0KA 4C0NA 4C0ZA 4C12A 4C1NK 4C1SA 4C1WA 4C1YA 4C24A 4C2FA 4C2LA 4C2MI 4C47A 4C4AA 4C5CA 4C5KA 4C5WA 4C6AA 4C6EA 4C6FA 4C6SA 4C76A 4C7AA 4C7GA 4C89A 4C8DA 4C8HA 4C93A 4C97A 4C9BB 4C9SA 4C9XA 4C9YA 4CA1A 4CADC 4CAHB 4CBCA 4CBEA 4CBHA 4CBPA 4CBUG 4CC2A 4CC9B 4CCKA 4CCVA 4CCZA 4CD5A 4CD8A 4CDJA 4CDPA 4CE8A 4CEMA 4CFPA 4CG1A 4CGKA 4CGOA 4CGUB 4CHDA 4CHIA 4CHMA 4CI7A 4CI8A 4CI9A 4CIDA 4CIHA 4CIJA 4CITA 4CJ0A 4CJDA 4CLCA 4CLLA 4CLQA 4CMRA 4CNGA 4CNKA 4CNNA 4CO6A 4COFA 4COGA 4CP6A 4CQHA 4CRHA 4CRUA 4CRUB 4CS4A 4CS9A 4CSSA 4CT3A 4CU4A 4CVBA 4CVNE 4CVOA 4CVQA 4CVRA 4CW5A 4CXPA 4CY8A 4CY9A 4CYFA 4CZGA 4CZXA 4CZXB 4D04A 4D0NB 4D0PA 4D1JA 4D2IA 4D5BA 4D5TA 4D6GA 4D6KA 4D6QA 4D6VA 4D6ZA 4D7CA 4D7EA 4D8BA 4D8MA 4D9BA 4D9IA 4D9SA 4DA2A 4DCAA 4DCMA 4DD5A 4DDPA 4DEVA 4DEYA 4DGFA 4DGUA 4DI9A 4DIDB 4DIMA 4DJAA 4DJDD 4DK2A 4DKJA 4DKKA 4DLHA 4DLOA 4DLQA 4DM5A 4DMGA 4DMIA 4DMVA 4DNHA 4DNYA 4DO4A 4DO7A 4DOIA 4DOOA 4DOXA 4DOYA 4DQ6A 4DQ9A 4DQAA 4DQZA 4DRIB 4DT5A 4DTHA 4DUIA 4DUQA 4DV8A 4DVGB 4DW1A 4DWDA 4DWEA 4DWLA 4DWRA 4DWRB 4DXTA 4DYLA 4DYNA 4DYOA 4DZ4A 4DZIA 4DZOA 4E0AA 4E0GA 4E15A 4E1SA 4E29A 4E2XA 4E3EA 4E3XA 4E3YA 4E40A 4E45E 4E4RA 4E4WA 4E5VA 4E5XG 4E6NA 4E6WA 4E6ZA 4E72A 4E74A 4E8UA 4E8YA 4E9SA 4E9XA 4EACA 4EADA 4EAEA 4EATA 4EBBA 4EBGA 4EDPA 4EE6A 4EFPA 4EGCB 4EGDA 4EGUA 4EGVA 4EHSA 4EHXA 4EI0A 4EI7A 4EICA 4EIJA 4EIUA 4EJQA 4EJRA 4EJYA 4EKFA 4EKXA 4EL6A 4EMEA 4EMNA 4EMOA 4EMTA 4ENEA 4ENFA 4EO0A 4EO7A 4EOGA 4EOZB 4EP4A 4EPCA 4EPSA 4EQ8A 4EQAC 4EQBA 4EQLA 4EQQA 4EQSA 4ER8A 4ERNA 4ERYA 4ES8A 4ESFA 4ESMA 4ESWA 4EU9A 4EUOA 4EV1A 4EVUA 4EVWA 4EWCA 4EXKA 4EXOA 4EXQA 4EXTA 4EYSA 4EYZA 4EZGA 4F01A 4F06A 4F0QA 4F1VA 4F27A 4F2DA 4F2ME 4F3NA 4F43A 4F4WA 4F52E 4F7GB 4F87A 4F8LA 4FA8A 4FA8E 4FBIA 4FBJA 4FBWA 4FC3E 4FC5A 4FCAA 4FCHA 4FCZA 4FDBA 4FDTA 4FDZA 4FFBC 4FFLA 4FGQA 4FGWA 4FHRA 4FINA 4FIXA 4FKMA 4FLBA 4FLEA 4FMPA 4FNVA 4FO0A 4FPRA 4FQNA 4FSDA 4FTBA 4FTFA 4FUVA 4FVGA 4FVQA 4FWVA 4FX5A 4FXIA 4FXQA 4FXWB 4FYYB 4FZ4A 4FZLA 4G0AA 4G0IA 4G0RA 4G0XA 4G10A 4G1IA 4G1OA 4G1QB 4G22A 4G26A 4G29A 4G2SA 4G38A 4G3FA 4G3HA 4G3NA 4G4GA 4G4SO 4G4SP 4G54A 4G55A 4G5HA 4G68A 4G6DB 4G6HA 4G6UA 4G79A 4G7NA 4G7XB 4G8TA 4G9PA 4GA2A 4GAXA 4GBMA 4GC0A 4GDZA 4GEHA 4GEIA 4GEYA 4GGJA 4GGVA 4GHKA 4GHNA 4GIMA 4GIOA 4GIPA 4GIWA 4GIZC 4GJ4A 4GJRA 4GJZA 4GKHA 4GLKA 4GM6A 4GMFA 4GMQA 4GMUA 4GNRA 4GOUA 4GPSA 4GQ1A 4GQBB 4GQOA 4GQZA 4GS1A 4GS3A 4GS5A 4GT8A 4GT9A 4GU5A 4GUCA 4GVBB 4GVFA 4GVQA 4GWBA 4GWGA 4GX0A 4GX7A 4GX8A 4GXTA 4GXWA 4GYOA 4GYTA 4GYVA 4GZ1A 4GZCA 4GZKA 4H03A 4H14A 4H18A 4H1BA 4H27A 4H2WA 4H3SA 4H3TA 4H3VA 4H3WA 4H40A 4H4DA 4H4NA 4H4VA 4H59A 4H5BA 4H5IA 4H5SA 4H61A 4H62Q 4H6CA 4H6QA 4H6XA 4H7LA 4H7WA 4H7YA 4H8EA 4H9NC 4HA6A 4HA7A 4HB9A 4HBQA 4HBSA 4HC9A 4HCWA 4HD5A 4HDDA 4HDOA 4HE6A 4HEIA 4HFVA 4HGXA 4HH3C 4HH8A 4HHJA 4HHOA 4HHRA 4HHVA 4HI4A 4HI6A 4HIKA 4HJ1A 4HJHA 4HKHA 4HKJD 4HL2A 4HLBA 4HLSA 4HMSA 4HNEA 4HNLA 4HORA 4HPMB 4HQ1A 4HR3A 4HR9A 4HRNC 4HRVA 4HS2A 4HSCX 4HSPA 4HSSA 4HSTB 4HT3A 4HT3B 4HT5A 4HTEA 4HTGA 4HU2A 4HUQS 4HUQT 4HV4A 4HVKA 4HVMA 4HW6A 4HWCA 4HWMA 4HWVA 4HY3A 4HY4A 4HYLA 4HYQA 4HZ9B 4HZOA 4I0OA 4I0UA 4I0XA 4I0XB 4I16A 4I1FA 4I1KA 4I1LA 4I1OB 4I2AA 4I2OA 4I3MA 4I4CA 4I4NA 4I4OA 4I4TA 4I4TE 4I4TF 4I5TA 4I66A 4I68A 4I6JB 4I6MA 4I6MB 4I6VA 4I6YA 4I71A 4I79A 4I79B 4I84A 4I86A 4I8IA 4I8OA 4I90A 4I93A 4I96A 4I99C 4I9OA 4I9XA 4IA6A 4IAJA 4IAUA 4IC3A 4IC4A 4IC9A 4ICGC 4ICVA 4ID9A 4IDHA 4IDOA 4IE5A 4IEFA 4IEJA 4IEUA 4IFAA 4IG1A 4IHQA 4II1A 4IIKA 4IILA 4IIWA 4IIYA 4IJAA 4IJNA 4IJYA 4IKDA 4IKNA 4IKVA 4IL7A 4ILLA 4IM7A 4IN0A 4INAA 4INDA 4INOA 4INWA 4INZA 4IOXA 4IOYX 4IPIA 4IPUA 4IQBA 4IQZA 4IRFA 4IRTA 4IRVA 4ISBA 4ISVB 4IT6A 4ITJA 4ITQA 4ITRA 4IU3B 4IUJA 4IUPA 4IUSA 4IW7A 4IWNA 4IWXA 4IX1A 4IX3A 4IX7A 4IYJA 4IYMA 4IZ7B 4IZHA 4IZUA 4IZXA 4J05A 4J0DA 4J0WA 4J1OA 4J1PA 4J1QA 4J1VA 4J27A 4J2CA 4J2GA 4J32A 4J33A 4J37A 4J42A 4J4HA 4J4ZA 4J5RA 4J6OA 4J73A 4J7HA 4J7NA 4J7OA 4J7QA 4J8LA 4J8SA 4J91A 4J9YB 4JA8A 4JB7A 4JBDA 4JBEA 4JBUA 4JCMA 4JD9A 4JDEB 4JDNA 4JDUA 4JDXA 4JEMA 4JF8A 4JG5A 4JG9A 4JGIA 4JGLA 4JGWA 4JHKA 4JHLA 4JHMA 4JHNA 4JIFA 4JIMA 4JIUA 4JJAA 4JJJA 4JJOA 4JL5A 4JLCA 4JLEA 4JMDA 4JMQA 4JMUA 4JN3A 4JN7A 4JN9A 4JNDA 4JNHA 4JO0A 4JO7A 4JOBA 4JOIC 4JOQA 4JP0A 4JP6A 4JPHA 4JPNA 4JPQA 4JPRA 4JQFA 4JQPA 4JQRA 4JR6A 4JR9A 4JRAC 4JRFA 4JRLA 4JS1A 4JS8A 4JTIA 4JTMA 4JVCA 4JVSA 4JX0A 4JX2A 4JXUA 4JZJC 4K0DA 4K0NA 4K12A 4K12B 4K1CA 4K1PA 4K22A 4K36A 4K3ZA 4K51A 4K5SA 4K6LG 4K6NA 4K70A 4K7BA 4K7CA 4K7JA 4K82A 4K8WA 4K90A 4K92A 4KA7A 4KALA 4KBXA 4KDDA 4KEMA 4KF8A 4KF9A 4KFUA 4KGDA 4KGHA 4KH8A 4KH9A 4KHBA 4KIAA 4KJMA 4KK0A 4KK7A 4KKRA 4KKZA 4KL0A 4KM6A 4KMDA 4KNAA 4KOPA 4KP1A 4KPPA 4KQ7A 4KQ9A 4KQAA 4KQDA 4KQIA 4KQPA 4KQWA 4KRGA 4KRRA 4KRXA 4KS9A 4KSNA 4KT3A 4KT3B 4KT5C 4KT6A 4KTIA 4KTWA 4KTYA 4KUIA 4KV2A 4KV7A 4KV9A 4KW3A 4KWDA 4KWYA 4KXVA 4KYPA 4KZPA 4KZSA 4L0CA 4L0JA 4L0KA 4L0RA 4L0VA 4L1NA 4L2HA 4L2IA 4L2WA 4L3NA 4L3RA 4L3UA 4L4EA 4L4QA 4L63A 4L68A 4L6UA 4L77A 4L7AA 4L7GA 4L8JA 4L8KA 4L8PA 4L9BA 4L9EA 4L9PA 4L9PB 4LA2A 4LANA 4LB0A 4LB8A 4LBAA 4LBUA 4LCLA 4LCTA 4LD1A 4LDAA 4LDVA 4LE3A 4LEBA 4LERA 4LF0A 4LG3A 4LG8A 4LGCA 4LGJA 4LH6A 4LHFA 4LHSA 4LITA 4LIXA 4LJ0A 4LJ6A 4LJ9A 4LJOA 4LJSA 4LL6A 4LL7A 4LLDB 4LLEA 4LMHA 4LMOA 4LMYA 4LNSA 4LO6B 4LOXA 4LP8A 4LPIA 4LPQA 4LPSA 4LQ6A 4LQ8A 4LQKA 4LR4A 4LRJA 4LRLA 4LRTA 4LRTB 4LRZE 4LS9A 4LSDA 4LSWA 4LT5A 4LTTA 4LTYA 4LTYC 4LUAA 4LUNU 4LV5A 4LV5B 4LV8A 4LVFA 4LVIA 4LW8A 4LWUA 4LX2A 4LXRJ 4LY1A 4LY4A 4LYAA 4LYPA 4LZJA 4M0NA 4M0QA 4M0WA 4M1GA 4M1GH 4M1HA 4M1XA 4M23A 4M2BA 4M2MA 4M37A 4M3PA 4M51A 4M5EA 4M5RA 4M66A 4M73A 4M7XA 4M82A 4M83A 4M88A 4M8AA 4M8RA 4M91A 4MAAA 4MAKA 4MAMA 4MAXA 4MB7A 4MBOA 4MBSA 4MBYA 4MC3A 4MCJA 4MCOA 4MCWA 4MD5B 4MDAA 4MDYA 4ME2A 4ME7E 4MEAA 4MERA 4MESA 4MEWA 4MFIA 4MFKA 4MFUA 4MGQA 4MHCA 4MHLA 4MI7A 4MIJA 4MIWA 4MIXA 4MIYA 4MJ2A 4MJ7A 4MJFA 4MJGA 4MJSB 4MKXA 4ML1A 4MLMA 4MM2A 4MMOA 4MMSA 4MN4C 4MNCA 4MNNA 4MNRA 4MO0A 4MO1A 4MP8A 4MPCA 4MPTA 4MQDA 4MQWX 4MRTC 4MS4B 4MSXA 4MT4A 4MTEA 4MTMA 4MU6A 4MU9A 4MUBA 4MUOA 4MUQA 4MUVA 4MV4A 4MVEA 4MVTA 4MXTA 4MYKA 4MYVA 4MZ2A 4MZ7A 4MZAA 4MZCA 4MZJA 4MZVA 4MZYA 4N01A 4N02A 4N04A 4N0HA 4N0HB 4N0LA 4N0NA 4N0RA 4N0TA 4N13A 4N1IA 4N1YA 4N2PA 4N2XA 4N3PA 4N3SA 4N3YB 4N49A 4N4JA 4N4PA 4N4UA 4N5BA 4N5HX 4N6CA 4N6DA 4N6KA 4N6OA 4N6OB 4N6QA 4N74A 4N75A 4N7QA 4N7RA 4N7RC 4N7WA 4N8GA 4N8PA 4N9WA 4N9XA 4NADA 4NAOA 4NARA 4NB5A 4NBPA 4NBXA 4NC7A 4NCKA 4NCXA 4NDNE 4NDSA 4NE2A 4NE3A 4NE3B 4NESA 4NF0A 4NF7A 4NFAA 4NFUB 4NG0A 4NG2E 4NGDA 4NHBA 4NHEA 4NHRA 4NI6A 4NJ6A 4NK2A 4NK6A 4NKBA 4NKGB 4NKPA 4NKRA 4NLCA 4NLMA 4NM6A 4NMIA 4NMYA 4NN2A 4NN5A 4NN5B 4NNOA 4NNZA 4NOFA 4NOGA 4NONA 4NOOB 4NPLA 4NPTA 4NPUA 4NQ0A 4NQFA 4NQIA 4NRDA 4NRHB 4NS5A 4NSDA 4NSSA 4NSVA 4NT1A 4NTDA 4NTKA 4NTQB 4NU5A 4NUAA 4NURA 4NUUA 4NUXA 4NV0A 4NV4A 4NWBA 4NWYA 4NXIA 4NXYA 4NYHA 4NYQA 4NZGA 4NZKA 4O06A 4O0CA 4O1IA 4O2HA 4O2TA 4O4FA 4O4OA 4O5AA 4O5JA 4O5VA 4O65A 4O6AA 4O6GA 4O6KA 4O6UA 4O7JA 4O87A 4O8SA 4O8VA 4O8WA 4O93B 4O9DA 4O9DB 4OA3A 4OAGA 4OBMA 4OBOA 4OBUE 4OCIA 4OCVA 4ODKA 4OE8A 4OE8B 4OEBA 4OFKA 4OFZA 4OGGA 4OGPA 4OH7A 4OHCA 4OHJA 4OHXA 4OI3A 4OI4A 4OIEA 4OJ8A 4OJXA 4OKEA 4OKOA 4OKZA 4OL4A 4OLEA 4OLOA 4OLSA 4OLTA 4OM8A 4OMBA 4OMFB 4OMGA 4OMVA 4ON1A 4ONQA 4OO3A 4OOGA 4OOXA 4OPCA 4OPMA 4OPWA 4OQ1A 4OQPA 4OQVA 4OR5A 4ORKA 4ORRA 4OTMA 4OTNA 4OTPA 4OU0A 4OU6A 4OU9A 4OUJA 4OUNA 4OUSA 4OV4A 4OV8A 4OVJA 4OVKA 4OVXA 4OVYA 4OW5A 4OW8A 4OWKA 4OWTA 4OXXA 4OY3A 4OY6A 4OYDB 4OZUA 4OZWA 4P04A 4P0GA 4P0TA 4P0ZA 4P17A 4P1NA 4P2VA 4P32A 4P37A 4P3AA 4P3FA 4P3HA 4P40A 4P5EA 4P5NA 4P5XA 4P6BA 4P78A 4P78C 4P7AA 4P7CA 4P7OA 4P82A 4P8NA 4P9FA 4P9GA 4PAGA 4PD0A 4PD6A 4PDYA 4PE3A 4PE6A 4PEDA 4PEIA 4PERA 4PEUA 4PEVA 4PF3A 4PF4A 4PFEA 4PFSA 4PFYA 4PG3A 4PGNA 4PGRA 4PHJA 4PHZC 4PIOA 4PJ2C 4PK9A 4PL0A 4PL3A 4PM4A 4PMOA 4PMXA 4PN0A 4PN6A 4PN7A 4PNEA 4PNOA 4PO6A 4PP4A 4PP8C 4PPUA 4PQ0A 4PQQA 4PQZA 4PS6A 4PSFA 4PSRA 4PSWA 4PSYA 4PT7A 4PTSA 4PUIA 4PUXA 4PVCA 4PVKA 4PW0A 4PW2A 4PW3D 4PWNA 4PXCA 4PXEA 4PXWA 4PXYA 4PY9A 4PYRA 4PZ0A 4PZ3A 4PZ7A 4PZOA 4PZUA 4Q05A 4Q0PA 4Q1QA 4Q1TA 4Q1ZA 4Q29A 4Q2LA 4Q2UA 4Q2WA 4Q3OA 4Q4FA 4Q4GX 4Q4W1 4Q4W2 4Q4W3 4Q51A 4Q5GA 4Q5WA 4Q62A 4Q63A 4Q68A 4Q6RA 4Q6TA 4Q6UA 4Q7FA 4Q7IA 4Q86A 4Q88A 4Q8GA 4Q8WA 4Q98A 4Q9TA 4QAMB 4QASA 4QB0A 4QBDB 4QBUA 4QC6A 4QDCA 4QDGA 4QEKA 4QFLA 4QFTA 4QFUA 4QGSA 4QIKA 4QITA 4QJKA 4QJVA 4QJVB 4QKYA 4QL0A 4QLAA 4QM6A 4QM9A 4QMFB 4QMGA 4QMHA 4QMKA 4QN8A 4QNUA 4QO5A 4QOZC 4QPMA 4QPWA 4QQDA 4QQGA 4QQRA 4QRHA 4QRNA 4QRSB 4QTCA 4QTDA 4QTJA 4QTNA 4QU6A 4QUVA 4QVRA 4QVUA 4QXBA 4QXBB 4QXLA 4QY7A 4QYTA 4R0CA 4R12A 4R1BA 4R1DA 4R1DB 4R1JA 4R1QA 4R23A 4R2FA 4R2XA 4R2YA 4R33A 4R3NA 4R3OG 4R3QA 4R4KA 4R4XA 4R6HA 4R6IA 4R6UA 4R6UC 4R6YA 4R75A 4R78A 4R7QA 4R7RA 4R80A 4R9FA 4R9IA 4R9OA 4RAAA 4RAPA 4RBRA 4RCJA 4RCTA 4RD4A 4RDBA 4RDQA 4REPA 4REVA 4RFAA 4RG1A 4RGIA 4RGLA 4RGPA 4RGWB 4RH0A 4RHJA 4RHOA 4RHPA 4RHWE 4RJWA 4RJZA 4RK2A 4RK4A 4RK6A 4RKSA 4RKUH 4RKUK 4RKUN 4RL6A 4RLZA 4RMLA 4RMMA 4RO3A 4ROCA 4ROQA 4RP3A 4RP9A 4RPLA 4RPMA 4RSCA 4RU0A 4RU1A 4RUWA 4RV2A 4RV5A 4RWRA 4RWRB 4RXLA 4RXMA 4RXTA 4RXXA 4RY1A 4RY8A 4RY9A 4RYRA 4RZ0A 4RZYA 4S1AA 4S1HA 4S1VD 4S28A 4S2VA 4S39A 4S3IB 4S3JA 4TJVA 4TKBA 4TKOB 4TKXL 4TM5A 4TMXA 4TPNA 4TPSB 4TQ3A 4TQRA 4TR3A 4TR6A 4TRHA 4TRKA 4TRTA 4TS4A 4TSHA 4TT0A 4TV7A 4TVOA 4TVSA 4TVVA 4TW5A 4TWBA 4TX3B 4TXDA 4TY0A 4TZ1A 4U04A 4U0OB 4U19A 4U1EG 4U1EI 4U1FA 4U3VA 4U3YB 4U4EA 4U4HA 4U4VA 4U5AA 4U5HA 4U5RA 4U5WA 4U63A 4U6UA 4U72A 4U7IA 4U7UA 4U7UB 4U7UE 4U8PA 4U98A 4U9OA 4U9PA 4U9UA 4UA8A 4UABA 4UADE 4UALA 4UC1A 4UCIA 4UD4A 4UDKE 4UE8A 4UEJA 4UF0A 4UF7A 4UHOA 4UMGA 4UMLA 4UOSA 4UPIA 4UQDA 4UQIA 4UQWA 4UQXA 4UR7A 4US5A 4USKA 4USRA 4UTUA 4UULB 4UUYA 4UV2A 4UVJA 4UVMA 4UW9A 4UWHA 4UWMA 4UXAA 4UXEA 4UXUA 4UY3A 4UYBA 4UYIA 4UZ1A 4UZ8A 4UZZB 4V03A 4V0KA 4V0PA 4V12A 4V1SA 4V24A 4V28A 4V2YA 4V33A 4V36A 4W4KA 4W4KB 4W4LC 4W4TA 4W7GA 4W7LA 4W7WA 4W82A 4W8BA 4W8KA 4W8PA 4WA0A 4WANA 4WATA 4WBDA 4WBTA 4WBYA 4WCKA 4WCXA 4WD1A 4WD3A 4WD8A 4WE2A 4WERA 4WI1A 4WIDA 4WIWA 4WJMA 4WK0A 4WKAA 4WKSC 4WLHA 4WMAA 4WMYA 4WPEA 4WU0A 4WUVA 4WVAA 4WW3A 4WW4B 4WWHA 4WY4D 4WZNA 4WZRA 4X1TA 4X1ZA 4X28A 4X28C 4X28D 4X33B 4X4WA 4X7KA 4X7RA 4X90A 4X9FA 4X9KA 4X9TA 4XB6C 4XB9A 4XCVA 4XDUA 4XE7A 4XEAA 4XEMA 4XFJA 4XFKA 4XFMA 4XGLA 4XGOA 4XGUD 4XGVD 4XHCA 4XJ5A 4XLGA 4XLYA 4XOHC 4XOTA 4XP7A 4XP9C 4XPZA 4XQ7A 4XQCA 4XRWA 4XSLB 4XUUA 4XUVA 4XVXA 4XWWA 4XYWA 4XZ6A 4XZ7A 4XZAA 4XZZA 4Y0HA 4Y1BA 4Y25A 4Y5UB 4Y68A 4Y7DA 4Y9TA 4YARA 4YC7B 4YD8A 4YDDB 4YDRA 4YFUA 4YHCA 4YHSA 4YJ6A 4YLRA 4YMKD 4YNVA 4YPOB 4YS4A 4YT2A 4YTBA 4YUCA 4YWTA 4YX6A 4YY0E 4YYCA 4YZGA 4YZIA 4YZKA 4YZOC 4YZZA 4Z0GA 4Z0YB 4Z13B 4Z67A 4Z6MA 4Z8TA 4Z8ZA 4Z9PA 4ZA9A 4ZASA 4ZB3A 4ZCDA 4ZDJA 4ZE8A 4ZELA 4ZEMA 4ZEOH 4ZFVA 4ZH0A 4ZHTB 4ZJNA 4ZKDA 4ZKQA 4ZL4A 4ZNMA 4ZOYA 4ZP0A 4ZPCA 4ZPXB 4ZR8A 4ZRPA 4ZRSA 4ZRXA 4ZS9B 4ZURA 4ZW2A 4ZW9A 4ZWND 4ZX2A 4ZXAW 5A07A 5A0JA 5A0YA 5A0YE 5A10A 5A1IA 5A1SC 5A3AA 5A61A 5A7GA 5A7VA 5A8CA 5A8JA 5A95B 5AB4B 5AFDA 5AFOA 5AGDA 5AH1A 5AHKA 5AHOA 5AJGA 5AJOA 5AM2A 5ANZA 5AP6A 5AWFA 5AWWY 5AX6A 5AX7A 5AY6B 5AYNA 5AYVB 5AZ3A 5AZBA 5AZPA 5B04B 5B04E 5B1QB 5B5RA 5BMNA 5BNZA 5BO7A 5BOVA 5BP3A 5BP8A 5BPKA 5BR4A 5BR7A 5BRAA 5BRHB 5BTOA 5BVAA 5BXAA 5BY7D 5BZ0A 5BZ3A 5BZAA 5C05B 5C0YA 5C1FB 5C2IB 5C2VB 5C2VC 5C40B 5C46E 5C55A 5C5CA 5C5HA 5C6DA 5C6PA 5C9SB 5CADA 5CAGA 5CCBB 5CCFA 5CCLA 5CD6A 5CDDB 5CDVA 5CE6A 5CECA 5CETA 5CFAA 5CG5A 5CGQB 5CHHA 5CHSA 5CIYA 5CKRA 5CKWB 5CO8A 5COTA 5COZA 5CPCA 5CPSA 5CQCA 5CQGA 5CQSA 5CR6D 5CR9A 5CU7A 5CWWB 5CX8A 5CXVA 5CZCA 5CZLA 5CZYA 5D2EA 5D3QA 5D6SA 5D91A 5DACB 5DAWA 5DC8B 5DCUB 5DDWD 5DEUA 5DGQB 5DI0B 5DJ1A 5DJ4D 5DLBA 5DMMA 5DN8A 5DNKB 5DP2A 5DQRE 5DSED 5DU9A 5DVIA 5DZ2A 5E3IA 5E3QA 5E71A 5E75A 5E8ZA 5E97A 5E9NA 5E9TD 5EC6A 5EFRA 5EFZF 5EGPB 5EJ8H 5EJRA 5EJYA 5EP9B 5EQNB 5ERMB 5ERQA 5ET1B 5EXEA 5EXEC 5EYBA 5EZ7A 5EZMA 5EZQA 5F2HA 5F2KA 5F2TA 5F86A 5F8CC 5FA1B 5FC1A 5FC2B 5FEWA 5FF5A 5FG0A 5FGUA 5FI3A 5FIAB 5FIWC 5FLWA 5FM7B 5FOEA 5FPWA 5FS8A 5FSHB 5FTBA 5FVND 5FX8U 5FXUB 5FYAB 5FZOA 5FZPB 5G0AA 5G0GA 5G1OD 5G3TB 5H9FA 5H9FI 5HAFA 5HAZA 5HDIB 5HDMB 5HHJA 5HIUD 5HIWA 5HJ9A 5HLRD 5HSXA 5HTXA 5HWNB 5HX0B 5HXAA 5HYAA 5HYZA 5HZLB 5I1UB 5I2CC 5I2HB 5I2MC 5I34B 5I39A 5I5HA 5I62A 5I6KA 5I90A 5IBVA 5IC7A 5IGOD 5IK4A 5IKJA 5IL2B 5IM2A 5IN3B 5IN4B 5IOJB 5ISUA 5IT0A 5IU4A 5IWSA 5IX8A 5IXPA 5IYZA 5IYZF 5IZTA 5J8QA 5J98C 5JAZB 5JDAA 5JEAC 5JEAH 5JGFA 5JIPB 5JJAB 5JLDA 5JM8F 5JQ3A 5JRJA 5JRYA 5JRZA 5JXMA 5JXZA 5K19A 5K2XA 5K4BA 5K5DC 5K5ZD 5K8CA 5KC8A 5KF9A 5KFZA 5KLAA 5KLPC 5KTGA 5KTNA 5L3DB 5L3XB 5L4QA 5L7JA 5L7ZA 5L9AA 5LB3E 5LF2B 5SUJA 7A3HA 7ODCA 8ABPA 9PAIA
